# Expression of CARD8 in human atherosclerosis and its regulation of inflammatory proteins in human endothelial cells

**DOI:** 10.1038/s41598-020-73600-4

**Published:** 2020-11-05

**Authors:** Geena V. Paramel, Glykeria Karadimou, Anna Göthlin Eremo, Liza U. Ljungberg, Ulf Hedin, Peder S. Olofsson, Lasse Folkersen, Gabrielle Paulsson-Berne, Allan Sirsjö, Karin Fransén

**Affiliations:** 1 Cardiovascular Research Centre, School of Medical Sciences, Faculty of Medicine and Health, Örebro University, 70182 Örebro, Sweden; 2 Laboratory of Immunobiology, Center for Bioelectronic Medicine, Department of Medicine, Karolinska Institute, Solna, Stockholm, Sweden; 3 Department of Clinical Research Laboratory, Faculty of Medicine and Health, Örebro University, Örebro, Sweden; 4 Department of Molecular Medicine and Surgery, Center for Molecular Medicine, Karolinska Institutet, Stockholm, Sweden; 5 Institute of Bioelectronic Medicine, Feinstein Institutes for Medical Research, Manhasset, NY USA; 6 Institute of Biological Psychiatry, Sankt Hans Hospital, Copenhagen, Denmark

**Keywords:** Mechanisms of disease, Chronic inflammation, Chemokines, Interleukins, Gene regulation in immune cells, Innate immunity, Diagnostic markers, Atherosclerosis

## Abstract

The Caspase activation and recruitment domain 8 (CARD8) protein is a component of innate immunity and overexpression of CARD8 mRNA was previously identified in atherosclerosis. However, very little is known about the regulation of CARD8 in endothelial cells and atherosclerosis. The aim of this study was to investigate CARD8 in the regulation of cytokine and chemokine expression in endothelial cells. Sections of human atherosclerotic lesions and non-atherosclerotic arteries were immunostained for CARD8 protein. Expression of CARD8 was correlated to mediators of inflammation in atherosclerotic lesions using Biobank of Karolinska Endarterectomies microarray data. The CARD8 mRNA was knocked-down in human umbilical vein endothelial cells (HUVECs) in vitro, followed by quantitative RT-PCR analysis and OLINK Proteomics. Endothelial and smooth muscle cells in arterial tissue expressed CARD8 and *CARD8* correlated with *vWF*, *CD163* and the expression of inflammatory genes, such as *CXCL1*, *CXCL6* and *PDGF-A* in plaque. Knock-down of CARD8 in HUVECs significantly altered proteins involved in inflammatory response, such as CXCL1, CXCL6, PDGF-A, MCP-1 and IL-6. The present study suggest that CARD8 regulate the expression of cytokines and chemokines in endothelial cells and atherosclerotic lesions, suggesting that CARD8 plays a significant role in endothelial activation.

## Introduction

Inflammation is a key component in the pathophysiology of atherosclerosis and involves numerous complex inflammatory cascades contributing to the inflammatory milieu in the atherosclerotic lesions. The caspase recruitment domain (CARD) was initially identified as a protein–protein interaction motif in the regulation of apoptosis^1^ and is also known for its function as scaffolding molecule to induce inflammation by activating NF-κB^2,3^. During the past decade, several CARD containing proteins, such as Nod1, Nod2, CARD10, Bcl10, CARD11 and CARD14 have been identified, to regulate activation of NF-κB^3^ via association with different adaptor proteins; CARD6, NOD1 and NOD2 recruit RIPK2, whereas CARD9, CARD10, CARD11 and CARD14 requires recruitment of BCL10 to activate NF-κB^4–9^.

One of the CARD containing proteins that has previously attracted focus is CARD8 (also known as TUCAN/CARDINAL/NDDP1). The CARD8 has in earlier studies been associated with a possible role in the NLRP3 inflammasome complex and has been found over-expressed in atherosclerotic lesions^10^. The *CARD8* gene has been extensively studied in relation to the C10X polymorphism, and several studies have suggested a possible association with various inflammatory diseases, although ambiguous results exist^11^. In cardiovascular disease (CVD), the minor variant of the C10X polymorphism has previously been associated with lower expression of *CARD8* mRNA levels in atherosclerotic plaques and to lower levels of CRP and MCP-1 in serum, indicating that *CARD8* may aggravate the atherosclerotic process by promoting inflammation^10^. However, the CARD8 regulatory mechanism is still not well understood. Unlike the other CARD proteins, CARD8 has been shown to inhibit NF-κB activation by interacting with IκB kinase complex (IKKγ)^12^. The CARD8 protein has also been suggested to inhibit the NOD2 dependent inflammatory response in epithelial cells and NLRP3 dependent IL-1β release in human monocyte derived macrophages^13,14^. On the other hand, earlier studies showed that CARD8 does not affect the IL-1β production and release in aortic smooth muscle cells (AoSMCs)^15^. Due to the fact that CARD8 is overexpressed in plaque, we hypothesize that CARD8 is important for the immune modulation in atherosclerosis. The aim of the present study is to examine the expression of CARD8 in human atherosclerotic lesion and to elucidate the role of CARD8 in the regulation of inflammatory proteins in endothelial cells and atherosclerotic lesions.

## Results

### CARD8 expression in healthy vessels and human carotid atherosclerotic lesions

**Figure 1:**
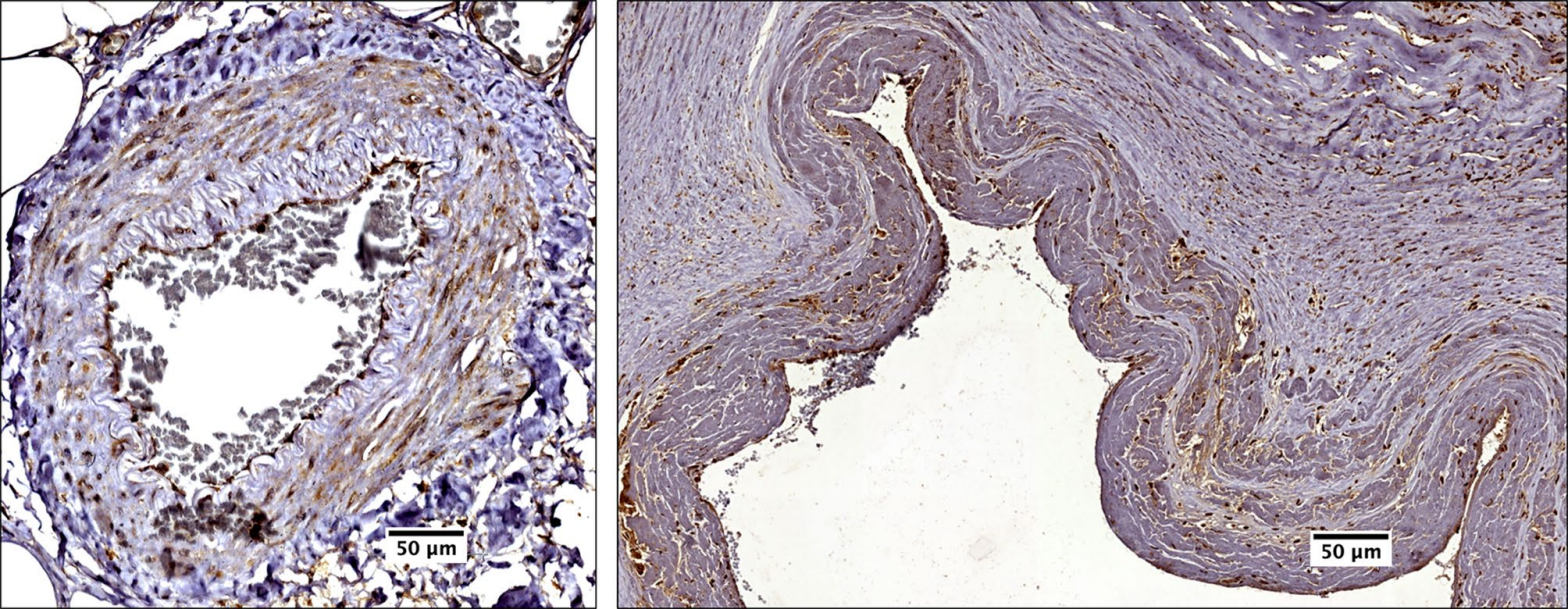
Immunostaining of CARD8 in non-atherosclerotic arteries. CARD8 expression was found in the endothelial layer of intima and smooth muscle cells in the media of non-atherosclerotic artery (Left: Artery from colon tissue, Magnification, × 30; Right: Popliteal artery, Magnification, × 10). Scale bar: 100 μm. Full images are available as Supplementary material.

**Figure 2:**
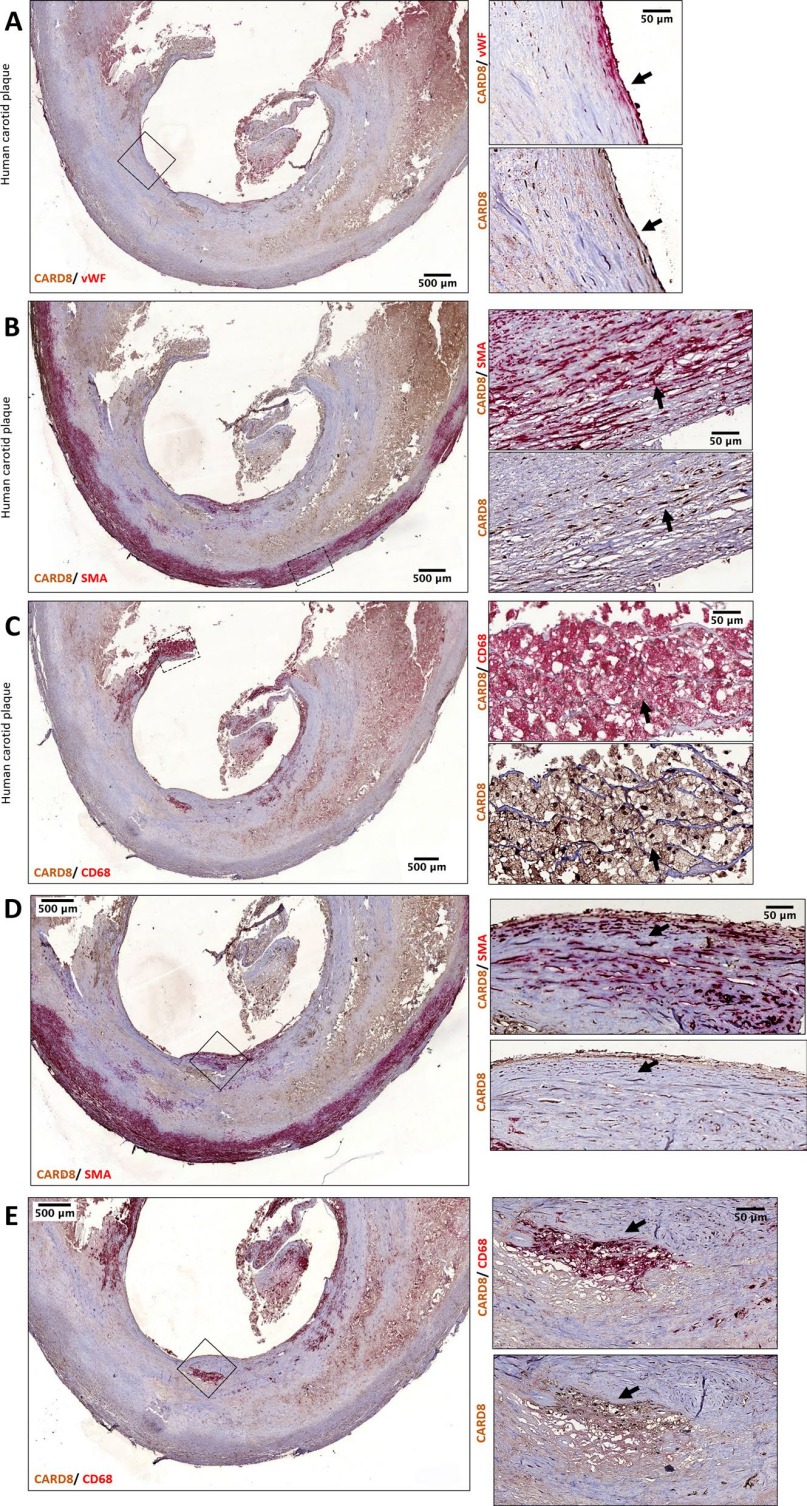
Representative image of immunostaining of human carotid atherosclerotic plaque. Expression of CARD8 staining (brown/DAB) was evident in endothelial cells is shown using the arrows (**A**), moderately in smooth muscle cells (red/SMA) (as shown with the arrow (**B**) and CD68 (red) positive macrophages (as shown with the arrow (**C**). Expression of CARD8 staining (brown/DAB) in the SMA (**D**) and CD68 (**E**) positive cells in the intimal region of lesions. Left Panel: Magnification, × 2 and Scale bar: 500 μm; Right Panel: Magnification, × 30 (D&E × 20) and Scale bar: 50 μm. Full images are available as Supplementary material.

**Figure 3:**
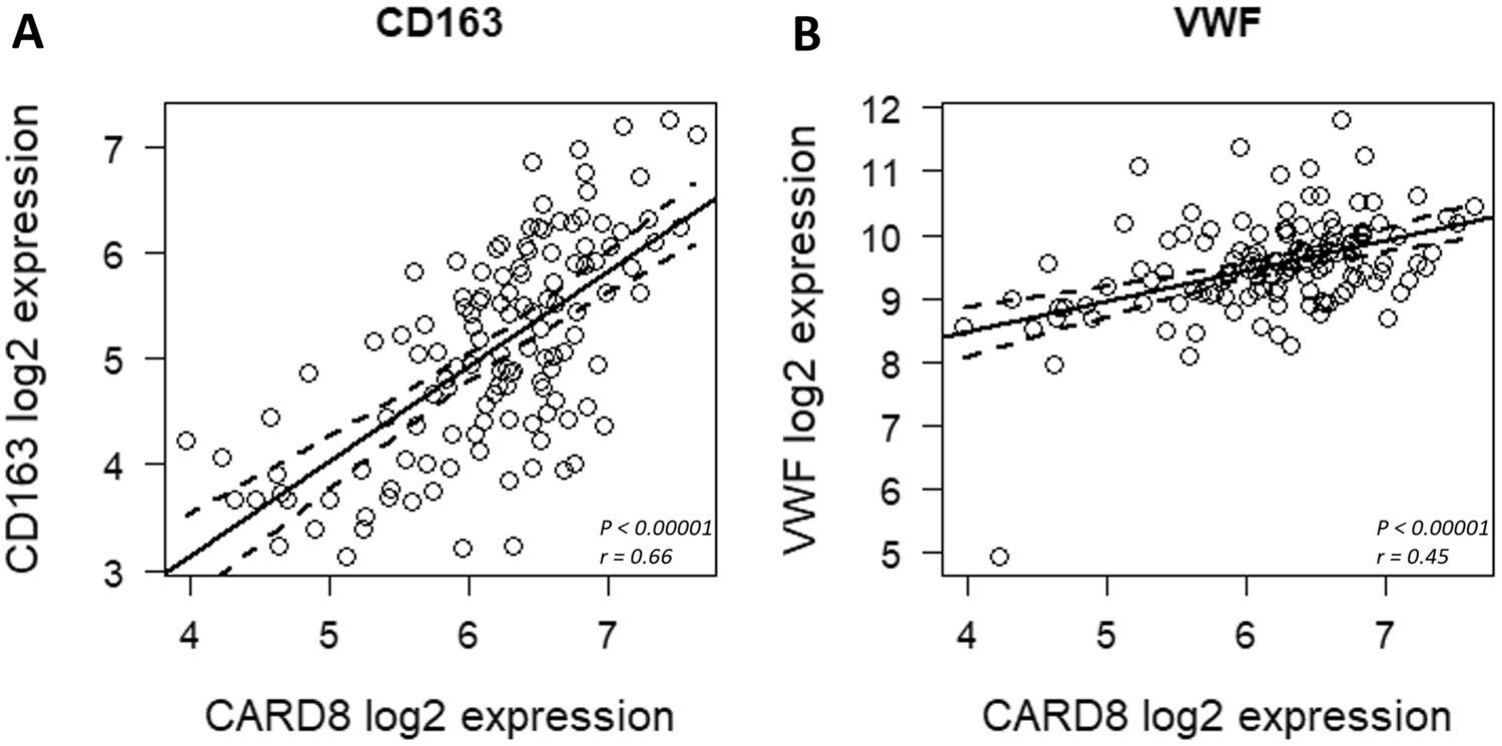
Correlation analysis using microarray data from BiKE study. *CARD8* expression positively correlated with the expression of *CD163* (**A**), and *VWF* (**B**), in human carotid atherosclerotic plaques. Solid line represent the trend line and the dashed lines represent the 95% confidence intervals for the trend lines.

We examined CARD8 expression in non-atherosclerotic artery and carotid lesions using immunohistochemistry. In the non-atherosclerotic vessels, CARD8 expression was primarily detected in the endothelial cells (Fig. 1). Also, smooth muscle cells (SMCs) in the tunica media stained positive for CARD8. In atherosclerotic carotid lesions, CARD8 was detected in the endothelial layer, smooth muscle cells and CD68 positive macrophages (Fig. 2), suggesting that the immune cells together with vascular cells contribute to the increased expression of CARD8 in the human atherosclerotic lesion. In addition, the CARD8 expression was identified in the intimal region of atherosclerotic carotid lesions and the expression was mainly localized in smooth muscle cells and CD68 positive cells. In microarray data from the BiKE study, expression of *CARD8* positively correlated with the macrophage marker *CD163* (r = 0.66; *p* < 0.00001) and von Willebrand factor (*VWF*; r = 0.45; *p* < 0.00001) endothelial marker (Fig. 3).

### Knock down efficiency and subcellular localization of CARD8 in HUVECs

**Figure 4:**
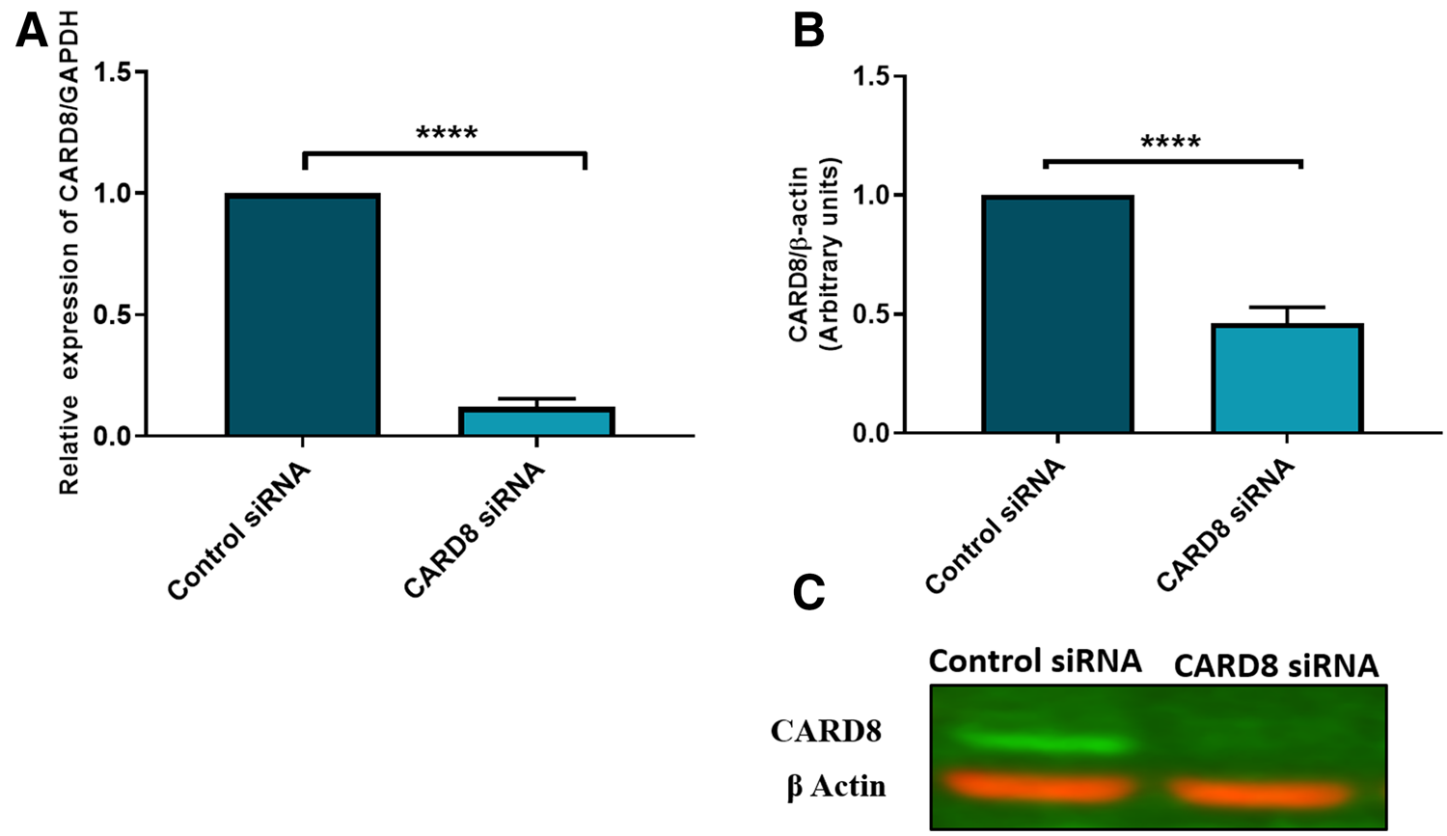
Expression of CARD8 mRNA and protein in control and *CARD8* knock down HUVECs. The knock down of *CARD8* significantly reduced the mRNA (**A**) and protein levels (**B**) of CARD8. Representative protein bands are shown in (**C**). The full image of (**C**) is shown as Supplementary data. Data is presented as mean ± SD for n = 3–6 in each group. *p* value ****p < 0.0001 is compared to control.

**Figure 5:**
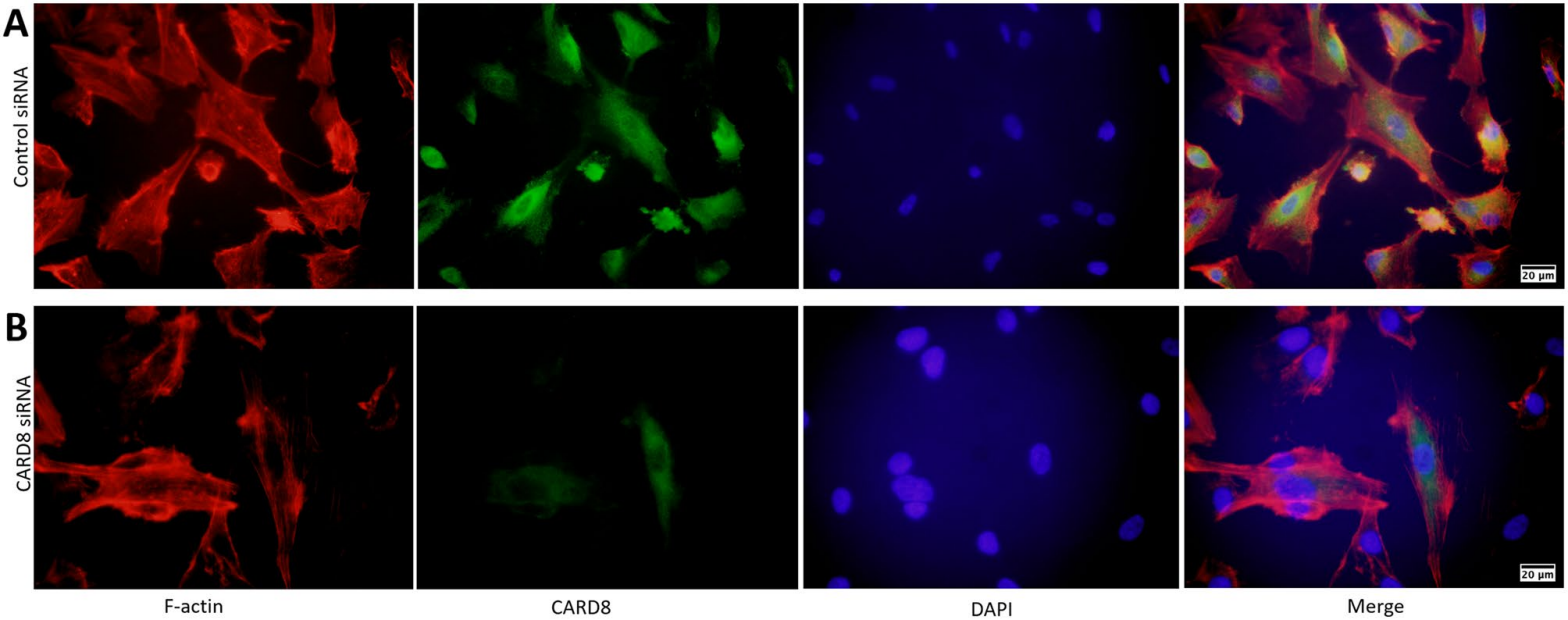
Subcellular localization of CARD8 in HUVEC. CARD8 expression in HUVECs treated with control/scramble siRNA (**A**) or CARD8 siRNA (**B**). CARD8 expression is indicated in *green*, f-actin in *red* and nucleus are stained *blue.* Magnification, × 40 and Scale bar: 20 μm. Full images are available as Supplementary material.

To investigate the role of CARD8 in HUVECs, the expression of *CARD8* gene was silenced in HUVECs using siRNA and the efficiency of knock down was measured in terms of CARD8 mRNA and protein. The levels of CARD8 mRNA and protein expression were significantly reduced (*p* < 0.0001 for mRNA and protein respectively; Fig. 4). The CARD8 expression was found both in the cytoplasm and nucleus of HUVECs (Fig. 5). A substantial reduction in CARD8 expression was evident in the CARD8 siRNA treated cells compared to the control siRNA treated cells, thereby also confirming the efficiency of CARD8 knock down in the CARD8 siRNA treated cells.

### CARD8 regulates the expression of inflammatory proteins in HUVECs

The effect of CARD8 knock-down on the expression of cytokines and chemokines was screened using three different OLINK Proseek Multiplex Assay panels; CVDII, CVDIII and Inflammation. Protein from lysate was analyzed on CVD II and CVD III panels, and protein from the culture medium was analyzed on Inflammation panel. In CVD II and CVD III panels, 46/92 and 49/92 proteins respectively, were detected in HUVECs (Supplementary Table S1). In total, 25 proteins out of 92 were detected in the cell medium using the Inflammation panel.

**Figure 6:**
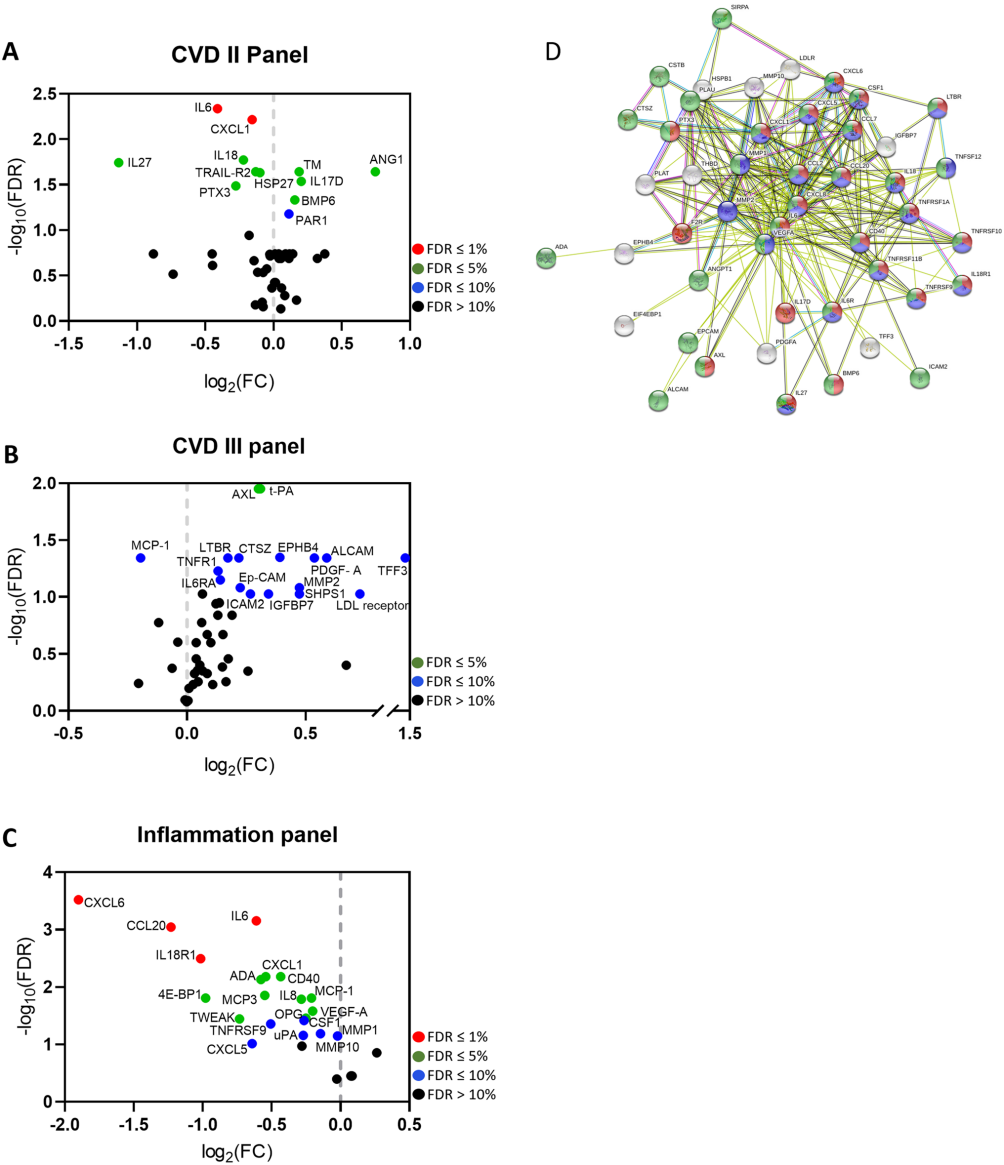
Differentially regulated proteins after CARD8 knock down (CARD8 KD) in HUVECs using Olink proteomics panels. Volcano plot displaying differential protein expression in the lysate (CVD II (**A**) and CVDIII (**B**) panels) and in the culture medium [Inflammation panel (**C**)]. Colors represent FDR levels (red, FDR ≤ 1%; green, FDR ≤ 5%; blue, FDR ≤ 10%; black, FDR > 10%). The labeled dots represent proteins that were differentially expressed in *CARD8* knock down versus control HUVECs (FDR ≤ 10%). (**D**) The protein–protein interaction network as analyzed by String software. Proteins in the STRING software corresponds to the following: TNFRSF10B = TRAIL-R2; F2R = PAR-1; THBD = TM; TNFRSF1A = TNF-R1; TNFSF12 = TWEAK; HSPB1 = HSP27; CCL2 = MCP-1; PLAT = tPA; CXCL8 = IL-8; EIF4EBP1 = 4E-BP1; CCL7 = MCP3; ANGPT1 = ANG1: LDLR = LDL receptor; SIRPA = SHPS1; TNFRSF11B = OPG; PLAU = uPA. The red, violet and green nodes represents proteins involved in inflammatory response, cytokine-mediated signaling pathway and immune system process respectively. The colored lines represent the different possible association between the proteins. A red line indicates the presence of fusion evidence; a green line indicates neighborhood evidence; a blue line indicates co-occurrence evidence; a purple line indicates experimental evidence; a yellow line indicates text-mining evidence; a light blue line indicates database evidence; and a black line indicates co-expression evidence.

Knock down of CARD8 in HUVECs significantly upregulated the levels of several proteins in the lysate, including ANG1, IL17-D, BMP6, TM, t-PA, AXL, PAR1, TFF3, LDL receptor, ALCAM, PDGF-A, MMP2, SHPS1, EPHB4, CTSZ, LTBR, TNFR1, IL-6RA, ICAM2, Ep-CAM and IGFBP7, all with FDR ≤ 10% (Fig. 6A,B, Supplementary Table S2).

None of the proteins in the medium were found upregulated in the Inflammation panel after *CARD8* knock down in HUVECs (FDR ≤ 10%; Fig. 6C). In addition to this, knock down of CARD8 in HUVECs resulted in reduction of IL-6, CXCL1, HSP27, TRAIL-R2, IL-18, PTX3, IL-27 and MCP-1 in the lysate (FDR ≤ 10%; Fig. 6A,B).

In the medium, IL-6, IL-18R1, CCL20, CXCL6, VEGF-A, OPG, MCP-1, IL-8, CD40, ADA, CXCL1, MCP3, TWEAK, 4E-BP1, MMP-1, MMP-10, CSF1, uPA, TNRFSF9 and CXCL5 were reduced after CARD8 knock down (FDR ≤ 10%; Fig. 6C).

Levels of inflammatory cytokines CXCL1, MCP-1 and IL-6 were significantly reduced both in the lysate and culture medium upon *CARD8* knock down. Next, we used the STRING software to derive an interaction network of significantly altered proteins. Gene Ontology analysis identified several enriched categories including inflammatory response, cytokine-mediated signaling pathway and immune system process pathways, suggesting the important role of CARD8 in mediating inflammation in the vessel wall (Fig. 6D).

### CARD8 regulates pro-inflammatory cytokines

**Figure 7:**
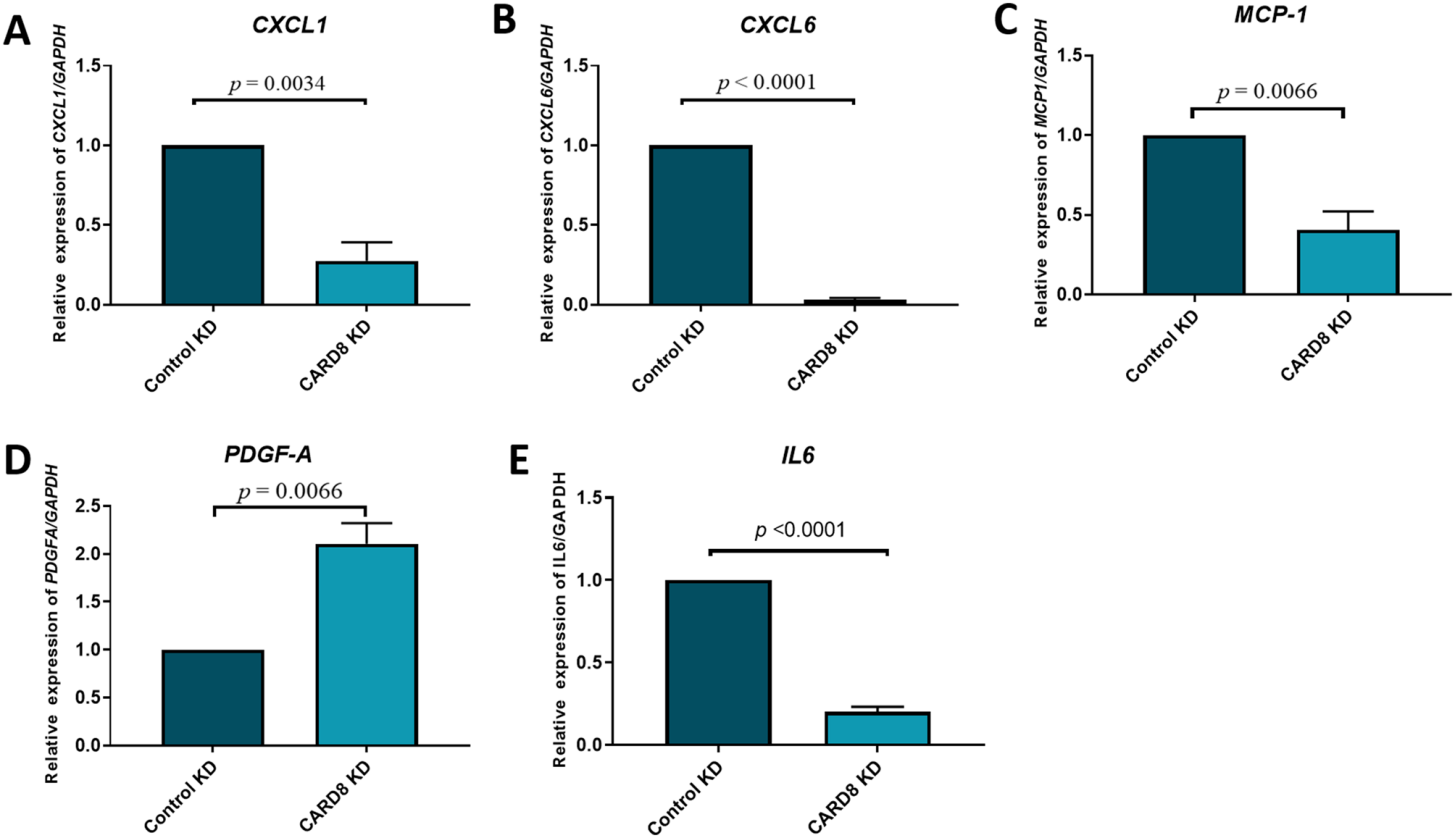
Gene expression levels of selected genes regulated by CARD8. The knock down of CARD8 significantly reduced the expression of *CXCL1* (*p* = 0.0034), *IL6* (*p* < 0.0001), *CXCL6* (*p* < 0.0001), and *MCP-1* (*p* = 0.0066), when compared to the control. The expression of *PDGF-A* (*p* = 0.0066) was significantly upregulated after the knock down of *CARD8* when compared to control. Data are representative of samples from 3 independent experiments and displayed as mean ± SD.

To investigate if *CARD8* knock down affects the transcription of inflammatory proteins, we examined the expression of some selective up- and downregulated proteins on the gene level. The mRNA levels of *CXCL1*, *IL6*, *CXCL6*, *MCP-1, ALCAM, PDGFA* and *IL-17D* were analyzed using qRT-PCR. The expression of *CXCL1*, *IL6, CXCL6* and *MCP-1* mRNAs were significantly reduced in *CARD8* knock-down cells compared to control cells (*CXCL1*, *p* = 0.0034; *IL6*, *p* < 0.0001; *CXCL6*, *p* < 0.0001; *MCP-1*, *p* = 0.0066; Fig. 7). The expression of *PDGFA* was significantly increased in the CARD8 knock-down cells as compared to the control cells (*PDGFA*, *p* = 0.0066; Fig. 7). The expression of *IL-17D* and *ALCAM* remained unaltered in the transcriptional level (data not shown). These findings indicate that CARD8 plays a role in the regulation of these inflammatory proteins.

### *CARD8* expression correlates with the mRNA expression of genes encoding inflammatory markers in the BiKE study

**Figure 8:**
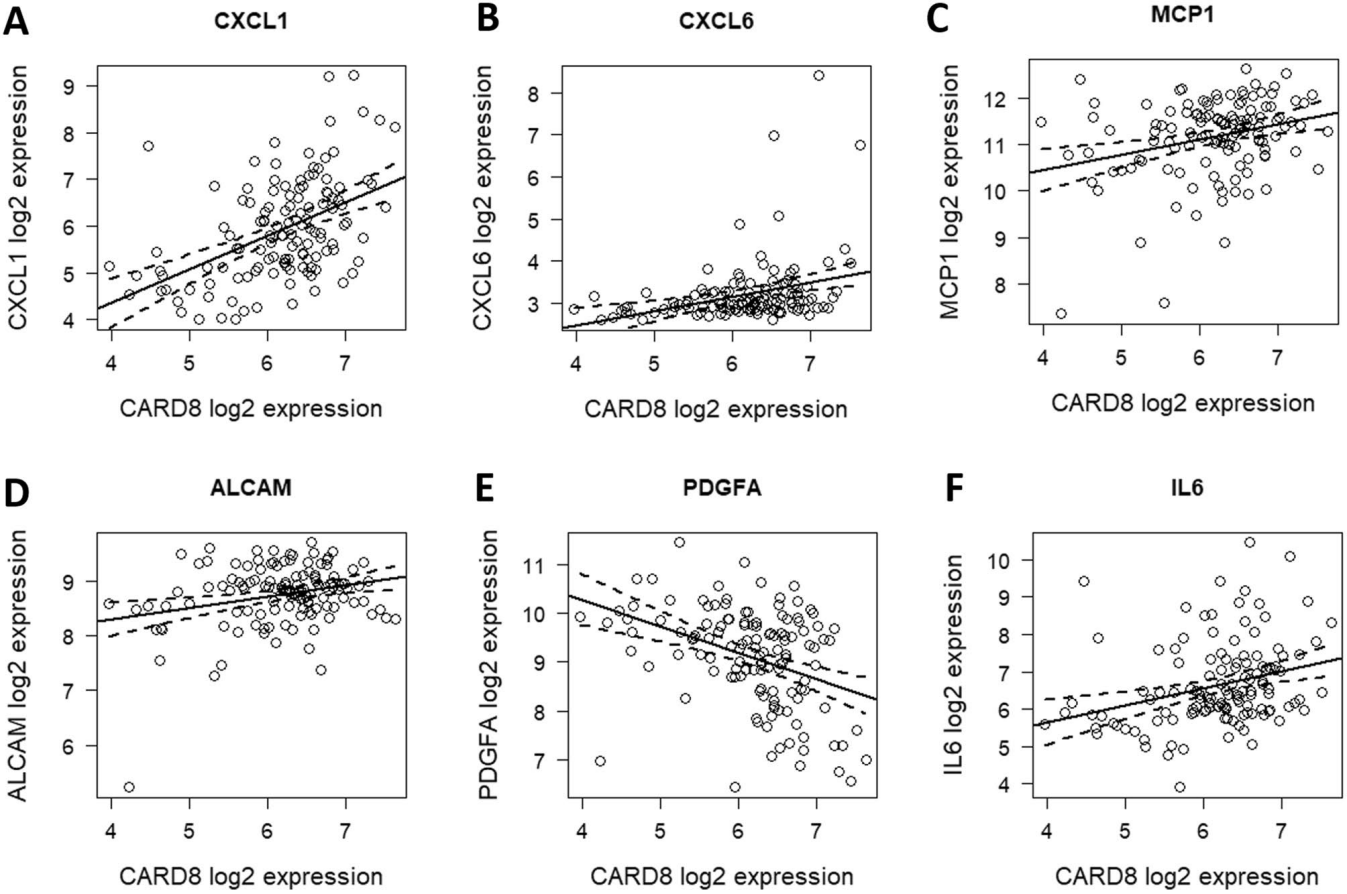
Correlation between gene expression of CARD8 and genes associated to inflammatory response and cell migration in human atherosclerotic lesions. *CARD8* correlated with (**A**) *CXCL1* (r = 0.48, *p* = 0.000000088); (**B**) *CXCL6* (r = 0.31, *p* = 0.0022); (**C**) *MCP-1* (r = 0.28, *p* = 0.011); (**D**) *ALCAM* (r = 0.26, *p* = 0.026); and (**E**) *PDGFA* (r = −0.38, *p* = 0.000062); (**F**) *IL6* (r = 0.29; *p* = 0.00082).

We further investigated the correlation of *CXCL1*, *IL6*, *CXCL6*, *MCP-1*, *ALCAM*, *PDGF-A* and *IL-17D* with *CARD8* in human carotid plaques using microarray data from the BiKE study. Expression of *CARD8* correlated significantly with *CXCL1* (r = 0.48; *p* = 0.000000088), *CXCL6* (r = 0.31; *p* = 0.0022), and *PDGFA* (r = −0.38, *p* = 0.000062; Fig. 8). Also, a weak correlation was found between the expression of *CARD8* and *MCP-1* (r = 0.28; *p* = 0.011), *IL6* (r = 0.29; *p* = 0.00082), and *ALCAM* (r = 0.26, *p* = 0.026; Fig. 8). Positive correlation was found between the expression of *CARD8* and *CXCL1* and *CARD8* and *CXCL6* respectively (Fig. 8). A weak positive correlation was also found between the expression of *CARD8* and *MCP-1, CARD8* and *IL6* and *CARD8* and *ALCAM* respectively (Fig. 8). The expression of *PDGFA* showed a negative correlation to the expression of *CARD8* (Fig. 8)*.* No significant correlation was found between the expression of *CARD8* and *IL-17D* (data not shown)*.*

## Discussion

In this study, we show that CARD8 protein is expressed in endothelial cells and SMCs of healthy and atherosclerotic vessels and that CARD8 may be an upstream regulator of several inflammatory cytokines and chemokines in both endothelial cells and human carotid plaques.

In a pilot study, we found that CARD8 is expressed in a variety of tissues such as brain, blood vessels, spleen, muscle, epidermis layer and hair follicles of skin (data not shown). The expression of *CARD8* in endothelial cells was confirmed in microarray data generated from the Oncomine 4.4 databank (www.oncomine.org; data not shown). In the present investigation, we found expression of CARD8 protein in endothelial cells and SMCs in non-atherosclerotic vessels. We also identified CARD8 protein expression in the intimal region in SMCs and CD68 positive cells. Previously we showed that *CARD8* mRNA expression was elevated in atherosclerotic lesions^10^. The present data complements the previous study by showing that in atherosclerotic lesions, immune cells, such as CD68 positive cells, endothelial cells and SMCs are the predominant cell types expressing CARD8 protein. We further correlated the expression of CARD8 in the human atherosclerotic lesion with the endothelial and macrophage markers with microarray data from the BiKE study. Consistent with the immunostaining, the expression of *CARD8* mRNA positively correlated with the mRNA expression of vWF (endothelial marker), and macrophage marker, CD163 in atherosclerotic lesions. The expression of CARD8 was localized both in the nucleus and cytoplasm of HUVECs, which was supported by a previous study where a similar subcellular localization of CARD8 was found in MCF-7 cells transfected with GFP tagged *CARD8* plasmid^12^. Our results are consistent with previously published analysis of gene expression pattern in atherosclerotic lesions showing that *CARD8* is upregulated in human atherosclerotic lesions and is significantly correlated to genes involved in inflammatory response, including *CCL2/MCP1* and *CD68* suggesting a role of CARD8 in mediating inflammatory markers by macrophages in the atherosclerotic lesion^16^.

Knowledge about the role of CARD8 in the regulation of inflammatory markers in endothelial cells and atherosclerosis is however limited. The CARD8 belongs to the bipartite CARDs, which consists of a CARD motif and one additional motif^17^. In contrast to other CARD containing proteins, initial studies showed that CARD8 is an inhibitor of NF-κB activation via physical interaction with the IκB kinase complex^12^. Previously, CARD8 was shown to regulate IL-1β release upon LPS and ATP stimulation in human-monocyte derived macrophages^13^. Contradictory, we have earlier shown that *CARD8* does not affect the IL-1β levels in AoSMCs^15^. Furthermore, knock down of CARD8 did not affect NF-κB activation in HUVECs (data not shown). In order to elucidate the role of CARD8 as a regulator of inflammation, we therefore silenced the *CARD8* gene to elucidate the role of CARD8 in endothelial cells. Knockdown of *CARD8* in HUVECs altered several proteins involved in inflammation and chemotaxis, such as CXCL1, IL-6, CXCL6, MCP-1, and PDGF-A. This was supported by the correlation of *CARD8* expression to *CXCL1*, *PDGFA* and *CXCL6* and a weak positive correlation to *MCP-1* and *IL6* in human atherosclerotic lesions in the BiKE study, which suggests a role of CARD8 in the regulation of these proteins in human atherosclerotic plaque.

Among the several CARD8 regulated proteins identified in the present study, there are several indications that CARD8 plays a role in atherosclerosis via its impact on downstream target genes. Knockdown of the chemokine CXCL1 has in previous studies been shown to reduce atherosclerosis in atherosclerosis-prone *LDLR*^*−/−*^ mice^18^. Furthermore, CXCL6 is involved in recruitment of neutrophils, but its role in CVD remains to be elucidated^19^. MCP-1 is a major chemotactic protein, that acts via CCR2 receptor and is induced by modified LDL and triggers adhesion of monocytes to the endothelium^20^. In the *ApoE* knock out mouse model, deletion of *MCP-1* leads to the reduction in atherosclerotic lesion size^21^, which may indicate on an important regulatory role for CARD8 in atherosclerosis via MCP-1. This is also consistent with our previous study showing elevated mRNA expression in atherosclerotic lesions and that lower expression of the truncated CARD8 rare variant is associated with lower levels of MCP-1 in plasma from patients with myocardial infarction^10^. Moreover, CARD8 regulates the expression of IL-6 in the endothelial cells. The cytokine IL-6 is known to regulate the synthesis of acute phase proteins, thereby contributing to the development of auto immune and chronic inflammatory disease^22^. IL-6 is also shown to induce the MCP-1 via JAK/STAT3 and PI3K/AKT pathways in human vascular endothelial cells^23^. Several studies indicate that blocking IL-6 signaling can be protective against inflammation^24^. Therefore, elevated levels of CARD8 in atherosclerosis may therefore contribute to increased inflammation in the pathogenesis of atherosclerosis by upregulation of CXCL1, CXCL6, IL-6 and possibly also MCP-1, may aggravate atherosclerosis.

In the present investigation, we also found upregulation of PDGF-A after knock down of *CARD8* in HUVECs and negative correlation to *CARD8* mRNA expression levels in atherosclerotic lesions in the BiKE study. Elevated levels of PDGF-A have been found in atherosclerotic lesions in *ApoE* mice^25^. In addition, PDGF-A is induced by oxidized low density lipoproteins and sheer stress in vascular smooth muscle cells and HUVECs respectively^26,27^. However, the role of CARD8 in the regulation of PDGF-A in atherosclerosis remains to be elucidated.

Several additional proteins were found reduced as a consequence of CARD8 knock-down, such as IL-8, TRAIL-R2, PTX3, CCL20, and MCP-3 and are involved in inflammation. Moreover, proteins like IL-8, PTX3, LDL-receptor and CCL20 are known to contribute to the development of atherosclerosis^28–30^. Our study also implicates on additional roles of CARD8, since proteins of hemostasis and wound healing, such as PAR-1, TM, tPA, uPA, TFF3 and AXL were found upregulated upon silencing of CARD8, suggesting the role of CARD8 to maintain hemostasis and wound healing process. CARD8 was also found to regulate the expression of cathepsin Z (*CTSZ*), a cysteine protease with exopeptidase activity regulating adhesion, migration and maturation of immune cells^31^. Similar to CARD8, the expression of *CTSZ* is significantly upregulated in human atherosclerotic lesion and was previously shown to significantly correlate to inflammatory markers, including *CCL2/MCP1* and *CD68* suggesting the possible role of *CTSZ* in inflammation^16^.

In conclusion, the current study shows that CARD8 protein is upregulated in atherosclerotic lesions and our data suggest that CARD8 may be involved in the regulation of the expression of cytokines and chemokines, such as CXCL1, CXCL6 and PDGF-A in vascular cells and atherosclerotic plaque, but also to other proteins related to inflammation, such as MCP-1 and IL-6. However, additional studies are warranted to elucidate the more precise mechanism of regulation of these proteins. Although we lack in vivo evidence of CARD8 as a regulator of inflammatory markers, the present study suggest a possible role for CARD8 as a novel mediator of inflammation in atherosclerotic lesions.

## Methods

### Immunohistochemistry

Carotid artery plaques were obtained from patients undergoing carotid endarterectomy at the Division of Thoracic and Cardiovascular Surgery, Örebro University Hospital, Sweden. Non-atherosclerotic arteries from colon tissue was obtained from the Division of Pathology, Örebro University Hospital and popliteal artery was obtained from the Karolinska Institute. The samples were used for immunostaining. The use of human atherosclerotic lesions and non-atherosclerotic arteries was ethically approved by Uppsala Regional Ethical Board (Dnr 2015/532) and the Karolinska Institute ethics committee (file number 02-147 and 2009/295-31/2) and informed written consent was obtained from all individuals. The study was ethically performed according to the guidelines of the Helsinki Declaration.

Atherosclerotic and non-atherosclerotic tissues were formalin fixed and paraffin embedded at the Department of Pathology, Örebro University Hospital, Sweden. The paraffin-embedded tissues were sectioned (4 μm) and subjected to deparaffinization and rehydration using Tissue Clear (Sakura, Alphen aan den Rijn, The Netherlands) and decreasing concentrations of ethanol. Epitope retrieval was performed in Diva decloaking buffer pH 6 (Biocare Medical, Pacheco, CA, USA) and Decloaking chamber (Biocare Medical) for 10 min at 110 °C. The staining was performed using primary antibodies against CARD8 (PAB0281; Abnova Corp, Taipei City, Taiwan; 1:1000/Nordic BioSite AB, Täby, Sweden), CD68 (NCL‐L‐CD68, Novacastra, Newcastle, UK; 1:50), VWF (M0616; Dako, Glostrup, Denmark; 1:50) and smooth muscle actin (SMA; M0851; Dako; 1:500) diluted in Da Vinci Green (Biocare Medical) and incubated for 1 h at room temperature. Subsequently, the antibodies were detected using the MACH 2 double stain HRP/AP polymer detection kit followed by visualization of CARD8 with 3,3″diaminobenzidine (DAB) and of SMA, CD68 and vWF using Warp Red. The tissue sections were counterstained with Mayer’s hematoxylin for 5 min at room temperature. Finally, the slides were dehydrated in increasing concentrations of ethanol prior to mounting with Pertex mounting medium (Histolab, Gothenburg, Sweden). The digital scanner Panoramic 250 Flash III (3DHistech, Budapest, Hungary) was used to scan the slides and micrographs were obtained from the Case Viewer using autosettings (Open source version 2.0 software; 3DHistech; https://www.3dhistech.com/).

### Microarray

Human atherosclerotic plaques from 126 patients undergoing endarterectomy for ischemic cerebrovascular disease were obtained from the Biobank of Karolinska Endarterectomies (BiKE), Karolinska University Hospital, Sweden. The sampling and the baseline characteristics of BiKE study have been described previously^32^. The study was approved by the Karolinska Institute ethics committee (file number 02-147 and 2009/295-31/2). Written consent was obtained from all participants and the study was ethically performed according to the guidelines of the Helsinki Declaration.

The human atherosclerotic plaques from the BiKE study were analyzed for gene expression via microarray using Affymetrix HG-U133 plus 2.0 Genechip arrays^32^. The raw data was processed using the robust microarray average algorithm and analyzed on a log2 scale, as recommended. Detailed methodology on the mRNA extraction and microarray protocol is mentioned elsewhere^32^. The BiKE data has been submitted to a public repository: https://www.omicsdi.org/dataset/arrayexpress-repository/E-GEOD-21545.

### Cell culture

Primary human umbilical vein endothelial cells (HUVECs; Thermo Fisher Scientific, Rockford, IL, USA), from pooled donors were cultured in VascuLife basal medium supplemented with VascuLife VEGF Life factors kit (Lifeline Cell Technology, GmbH, Troisdorf, Germany) in a humidified incubator with 5% CO_2_ at 37 °C. The cells between passages 4 and 8 were used in the experiment.

### Transfection using siRNA in HUVECs

The HUVECs (2 × 10^5^ cells/well) were plated in six-well plates and allowed to grow overnight and transfected with siRNA followed by incubation for 48 h. The transfection mixture comprised of CARD8 stealth RNAi (Cat. no. 53621349; Invitrogen, Carlsbad, CA, USA) or stealth RNAi siRNA Negative Control Med GC Duplex (Cat. no. 462001, Invitrogen) at a final concentration of 10 nM, and Lipofectamine 2000 (Cat. no. 11668-019, Invitrogen), diluted in Opti-MEM Reduced Serum Medium (Invitrogen) according to the manufacturer’s protocol.

### Subcellular localization of CARD8 in HUVECs

HUVECs were transfected with CARD8 or control siRNA by lipofection followed by incubation for 48 h. *CARD8* knock down and control HUVECs were washed gently with 1 × PBS and fixed with ice-cold 4% paraformaldehyde for 40 min at room temperature. Following the incubation, the cells were washed gently with PBS and incubated with ice-cold PBS containing 0.1% Triton-× 100 for 10 min. The cells were washed again with ice-cold PBS and blocked with 1% BSA in PBS containing 0.1% Triton-× 100 for 30 min at room temperature. After gentle rinse with ice-cold PBS, the cells were stained for CARD8 using CARD8 rabbit polyclonal antibody (1:500) diluted in PBS (Abnova, Taipei, Taiwan/Nordic BioSite) for 1 h at room temperature. The cells were washed again and incubated with Alexa Fluor 488 goat anti rabbit IgG (working concentration of 2 mg/ml, Invitrogen) at room temperature for 1 h. After rinsing with ice-cold PBS, the cells were stained for F-actin using Rhodamine phallodin (5U/200 μl) in the dark for 20 min. Cells were washed twice with PBS and the nucleus was stained using 4′,6′-diamidino-2-phenylindole hydrochloride (DAPI; Sigma, Deisenhofer, Germany) in the dark for 5 min. The cover slips were removed from the wells and mounted on to slides using mounting media and viewed under fluorescence microscope Olympus BX60 fluorescence microscope (Olympus Europe, Hamburg, Germany). Images were obtained with Olympus DP71 camera (Olympus Europe).

### RNA extraction and quantitative real-time PCR (qRT-PCR) analysis

Total RNA was extracted from *CARD8* knock down and control HUVECs using the E.Z.N.A Total RNA Kit I (Omega Bio-Tek, Norcross, GA, USA) in accordance to the manufacturer’s instructions. The cDNA was prepared using High Capacity cDNA reverse transcription kit (Thermo Fisher Scientific), and analyzed for the mRNA expression of chemokine CXC motif ligand 1 (*CXCL1*; Hs00236937)*,* chemokine CXC motif ligand 6 (*CXCL6*; Hs00605742)*,* monocyte chemotactic protein 1 (*MCP-1;* Hs00234140)*,* interleukin 6 (*IL6; Hs00174131*), platelet-derived growth factor, alpha (*PDGFA*; Hs00234994)*,* activated leukocyte cell adhesion molecule (*ALCAM;* Hs00977641) and peptidyl-prolyl *cis*–*trans* isomerase B (*PPIB*; Hs00168719) and interleukin-17D (*IL-17D*; Hs05007146) using TaqMan universal PCR master mix, TaqMan primers and probes and 7900HT Fast Real Time PCR (Thermo Fisher Scientific) according to the manufacturer’s instructions. The data were normalized relative to *PPIB* as endogenous control.

### OLINK Proseek Multiplex Assay

Cell lysates and culture medium from *CARD8* knock down and control HUVECs from three individual experiments were analyzed utilizing Cardiovascular II (CVDII), Cardiovascular III (CVDIII) and Inflammation panels. The panels contain a broad array of 92 established protein biomarkers each for CVD and inflammation (www.olink.com), via Olink Proseek Multiplex Assay using proximity extension assay (PEA) technology^33,34^ (Olink Proteomics, Uppsala, Sweden). Protein quantities were log2 transformed using Olink Wizard GenEx (MultiD Analyses, Gothenburg, Sweden). Gene ontology analysis was performed using the STRING software (version 11.0; https://string-db.org/) to derive network interaction between the significantly altered proteins.

### Statistical analysis

Pearson′s method was used to calculate correlation coefficients, and all correlations were adjusted for multiple comparisons using the Benjamini–Hochberg false-discovery rate (FDR) method. Experimental data are represented as mean ± SEM. Student’s T-test was used to analyze the statistical difference between two groups. A *p* value ≤ 0.05 was considered as the level of significance. For the statistical comparison of two groups, multiple T-test with FDR threshold of 1%, 5% and 10% (two-stage linear step-up procedure of Benjamini et al.^35^), with Q = 1%, Q = 5% and Q = 10%) were performed using GraphPad Prism (version 5.01; GraphPad Software, San Diego, CA, USA; https://www.graphpad.com/scientific-software/prism/).

## Supplementary information

Supplementary Information.
