# Author Correction: Expression of CARD8 in human atherosclerosis and its regulation of inflammatory proteins in human endothelial cells

**DOI:** 10.1038/s41598-021-88467-2

**Published:** 2021-04-19

**Authors:** Geena V. Paramel, Glykeria Karadimou, Anna Göthlin Eremo, Liza U. Ljungberg, Ulf Hedin, Peder S. Olofsson, Lasse Folkersen, Gabrielle Paulsson-Berne, Allan Sirsjö, Karin Fransén

**Affiliations:** 1 Cardiovascular Research Centre, School of Medical Sciences, Faculty of Medicine and Health, Örebro University, 70182 Örebro, Sweden; 2 Laboratory of Immunobiology, Center for Bioelectronic Medicine, Department of Medicine, Karolinska Institute, Solna, Stockholm, Sweden; 3 Department of Clinical Research Laboratory, Faculty of Medicine and Health, Örebro University, Örebro, Sweden; 4 Department of Molecular Medicine and Surgery, Center for Molecular Medicine, Karolinska Institutet, Stockholm, Sweden; 5 Institute of Bioelectronic Medicine, Feinstein Institutes for Medical Research, Manhasset, NY USA; 6 Institute of Biological Psychiatry, Sankt Hans Hospital, Copenhagen, Denmark

Correction to: *Scientific Reports* 10.1038/s41598-020-73600-4, published online 05 November 2020

The original version of this Article contained an error in the spelling of the author Gabrielle Paulsson-Berne, which was incorrectly given as Gabrielle Paulsson Berne. In addition, the author Gabrielle Paulsson-Berne was incorrectly indexed.

The original Article and accompanying Supplementary Information file have been corrected. The corrected Supplementary Information file is also linked to this correction notice.

## Supplementary Information

Supplementary Information.

