# Supplementary material for: Expression of CARD8 in human atherosclerosis and its regulation of inflammatory proteins in human endothelial cells
Source: Sci Rep. 2020 Nov 5;10:19108. doi: 10.1038/s41598-020-73600-4 (PMC7644683; doi:10.1038/s41598-020-73600-4)
Supplement: Supplementary file 1 — Supplementary Information. [file 41598_2020_73600_MOESM1_ESM.pdf]

Expression of CARD8 in human atherosclerosis and its regulation of inflammatory proteins in human endothelial cells

Geena V Paramel[1], Glykeria Karadimou[2], Anna Göthlin Eremo[3], Liza U Ljungberg[1], Ulf Hedin[4], Peder S Olofsson[2, 5], Lasse Folkersen [6], Gabrielle Paulsson-Berne[2], Allan Sirsjö [1], Karin Fransén[1*].

[1]Cardiovascular Research Centre, School of Medical Sciences, Faculty of Medicine and Health, Örebro University, Örebro, Sweden; [2]Laboratory of Immunobiology, Center for Bioelectronic Medicine, Department of Medicine, Solna, Karolinska Institute, Stockholm, Sweden; [3]Department of Clinical Research Laboratory, Faculty of Medicine and Health, Örebro University, Örebro, Sweden; [4]Department of Molecular Medicine and Surgery, Center for Molecular Medicine, Karolinska Institutet, Stockholm, Sweden; [5]Institute of Bioelectronic Medicine, Feinstein Institutes for Medical Research, Manhasset, New York, U.S.A; [6]Institute of Biological Psychiatry, Sankt Hans Hospital, Copenhagen, Denmark.

*** Corresponding author:** Karin Fransén, Cardiovascular Research Centre, School of Medical Sciences, Örebro University; 70182 Örebro, Sweden. ORCID: 0000-0002-9826-0462. Tel: +46-19-301408; Email: karin.h.franzen@oru.se

Short title: CARD8 – a regulator of inflammatory proteins

Key words: Atherosclerosis, Inflammation, NLRP3, CARD8, Inflammasome

Supplementary Table S1. Protein biomarkers available on the CVD II, CVDIII and Inflammation panels. Uncolored cells represents the proteins detected in HUVECs. The colored cells represents proteins that were below the detection level.

Inflammation panel (culture medium)	CVDII panel (Lysate)	CVD III panel (Lysate)
IL-8	BMP-6	TNFRSF14
VEGF-A	ANG-1	LDL receptor
MCP-3	ADM	IL-17RA
OPG	PlGF	TNF-R2
LAP TGF-beta-1	IL-4RA	EPHB4
uPA	SRC	OPG
IL-6	IL-6	ALCAM
MCP-1	TNFRSF10A	TFF3
CXCL11	STK4	SELP
CXCL1	IDUA	CSTB
MMP-1	TNFRSF11A	MCP-1
IL-18R1	PAR-1	Gal-3
CXCL5	TRAIL-R2	GRN
HGF	TIE2	BLM hydrolase
MMP-10	TF	PLC
Flt3L	IL1RL2	LTBR
CXCL6	PDGF subunit B	FABP4
CXCL10	IL27	TFPI
4E-BP1	IL-17D	PAI
CD40	CXCL1	TR
TNFRSF9	Gal-9	TNFRSF10C
TWEAK	SCF	GDF-15
CCL20	IL-18	CXCL16
ADA	SOD2	IL-6RA
CSF-1	FS	TR-AP
IL-1 alpha	GLO1	Ep-CAM
SCF	CD84	AP-N
GDNF	PAPPA	AXL
CDCP1	DECR1	IL-1RT1
CD244	TM	MMP-2
IL-7	HO-1	FAS
IL-17C	IL16	U-PAR
IL-17A	SORT1	CTSD
AXIN1	PTX3	JAM-A
TRAIL	PSGL-1	SHPS-1
IL-20RA	MMP-7	CASP-3
CXCL9	Dkk-1	uPA
CST5	LPL	ST2

IL-2RB	HB-EGF	t-PA
OSM	BNP	IGFBP-7
IL-2	PD-L2	CD93
TSLP	CTSL1	CTSZ
CCL4	TGM2	ICAM-2
CD6	HSP 27	PDGF subunit A
BNDF	NEMO	TNF-R1
IL-18	PARP-1	IGFBP-2
SLAMF1	FGF-21	vWF
TGF-alpha	RAGE	PECAM-1
MCP-4	CD40-L	IL-18BP
CCL11	SLAMF7	SELE
TNFSF14	ADAM-TS13	ITGB2
FGF-23	Protein BOC	MMP-9
IL-10RA	IL-1ra	IL2-RA
FGF-5	PRSS27	CD163
LIF-R	LOX-1	MEPE
FGF-21	GIF	Notch 3
CCL19	PIgR	TIMP4
IL-15RA	CTRC	CNTN1
IL-10RB	FGF-23	CDH5
IL-22 RA1	SPON2	TLT-2
PD-L1	GH	CCL24
Beta-NGF	SERPINA12	AZU1
TRANCE	REN	DLK-1
IL-12B	MERTK	SPON1
IL-24	TIM	MPO
IL-13	THBS2	RETN
ARTN	VSIG2	IGFBP-1
IL-10	AMBP	CHIT1
TNF	PRELP	CCL22
CCL23	XCL1	PSP-D
CD5	CEACAM8	PI3
MIP-1 alpha	CCL17	MB
IL-20	CCL3	TNFSF13B
SIRT2	IgG Fc receptor II-b	PRTN3
CCL28	ITGB1BP2	PCSK9
DNER	DCN	OPN
EN-RAGE	PRSS8	PGLYRP1
IL-33	AGRP	CPA1
IFN-gamma	GDF-2	Gal-4
FGF-19	FABP2	IL-1RT2
IL-4	THPO	CCL15

LIF	MARCO	CPB1
NRTN	GT	CHI3L1
MCP-2	MMP-12	SCGB3A2
CASP-8	ACE2	EGFR
CCL25	hOSCAR	COL1A1
CX3CL1	TNFRSF13B	PON3
NT-3	LEP	MMP-3
ST1A1	CA5A	RARRES2
STAMPB	CD4	KLK6
IL-5	VEGF-D	NT-pro BNP
TNFB	HAOX1	CCL16

Supplementary Table S2. Summary of differentially expressed proteins in the CARD8 knock down HUVECs according to OLINK proteomics data. Fold change in CARD8 knock down vs. Control knock down cells; significance at FDR ≤ 10%.

**CVDII Panel**			
Protein ID	Fold Change(log_2)	FDR	Protein name
IL6	-0,411	0,005	Interleukin-6
CXCL1	-0,157	0,006	C-X-C motiff chemokine
IL18	-0,220	0,017	Interleukin-18
IL27	-1,134	0,018	Interleukin-27
TRAIL-R2/TNFRSF10B	-0,131	0,023	TNF-related apoptosis-inducing ligand receptor 2
ANG1/ANGPT1	0,747	0,023	Angiopoietin-1
TM/THBD	0,188	0,023	Thrombomodulin
HSP27/HSPB1	-0,100	0,023	Heat shock 27 kDa protein
IL17D	0,203	0,029	Interleukin-17D
PTX3	-0,276	0,033	Pentraxin-related protein
BMP6	0,156	0,047	Bone morphogenetic protein 6
PAR1/F2R	0,114	0,066	Proteinase-activated receptor 1
**CVDIII Panel**			
Protein ID	Fold Change(log_2)	FDR	Protein name
t-PA/PLAT	0,310	0,011	Tissue-type plasminogen activator
AXL	0,301	0,011	Tyrosine-protein kinase receptor UFO
EPHB4	0,392	0,045	Ephrin type-B receptor 4
LTBR	0,172	0,046	Lymphotoxin-beta receptor
MCP-1/CCL2	-0,196	0,046	Monocyte chemotactic protein 1
TFF3	1,415	0,046	Trefoil factor 3
ALCAM	0,589	0,046	CD166 antigen
PDGF-A	0,537	0,046	Platelet-derived growth factor subunit A
CTSZ	0,219	0,046	Cathepsin Z
TNF-R1/TNFRSF1A	0,131	0,059	Tumor necrosis factor receptor 1
IL-6RA	0,140	0,071	Interleukin-6 receptor subunit alpha
Ep-CAM	0,224	0,083	Epithelial cell adhesion molecule
MMP-2	0,474	0,083	Matrix metalloproteinase-2
LDL receptor/LDLR	0,729	0,094	Low-density lipoprotein receptor
CSTB	0,065	0,094	Cystatin-B
IGFBP-7	0,344	0,094	Insulin-like growth factor-binding protein 7
ICAM-2	0,268	0,094	Intercellular adhesion molecule 2
SHPS-1/SIRPA	0,474	0,094	Tyrosine-protein phosphatase non-receptor type substrate 1
**Inflammation Panel**			
Protein ID	Fold Change(log_2)	FDR	Protein name
CXCL6	-1,901	0,0003	C-X-C motif chemokine 6
IL6	-0,610	0,0007	Interleukin-6
CCL20	-1,230	0,0009	C-C motif chemokine 20
IL18R1	-1,014	0,0032	Interleukin-18 receptor 1
CXCL1	-0,543	0,0066	C-X-C motif chemokine 1
CD40	-0,435	0,0066	CD40L receptor
ADA	-0,577	0,0074	Adenosine Deaminase
MCP3/CCL7	-0,549	0,014	Monocyte chemotactic protein 3

MCP1/CCL2	-0,211	0,0156	Monocyte chemotactic protein 1
4E-BP1/EIF4EBP1	-0,978	0,0156	Eukaryotic translation initiation factor 4E-binding protein 1
IL8/CXCL8	-0,285	0,0163	Interleukin-8
VEGF-A	-0,202	0,0262	Vascular endothelial growth factor A
OPG/TNFRSF11B	-0,251	0,0348	Osteoprotegerin
TWEAK/TNFSF12	-0,732	0,0359	Tumor necrosis factor (Ligand) superfamily, member 12
CSF1	-0,264	0,0382	Macrophage colony-stimulating factor 1
TNFRSF9	-0,506	0,0439	Tumor necrosis factor receptor superfamily member 9
MMP10	-0,146	0,065	Matrix metalloproteinase-10
uPA/PLAU	-0,270	0,0693	Urokinase-type plasminogen activator
MMP1	-0,021	0,0711	Matrix metalloproteinase-1
CXCL5	-0,641	0,0969	C-X-C motif chemokine 5

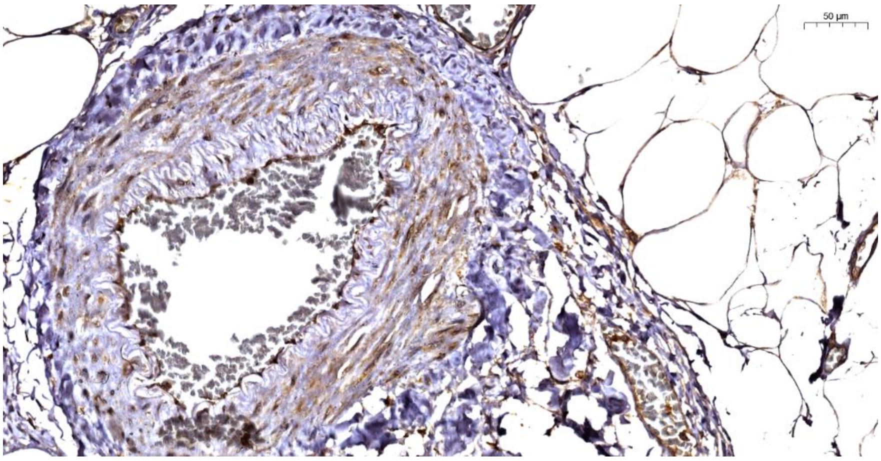

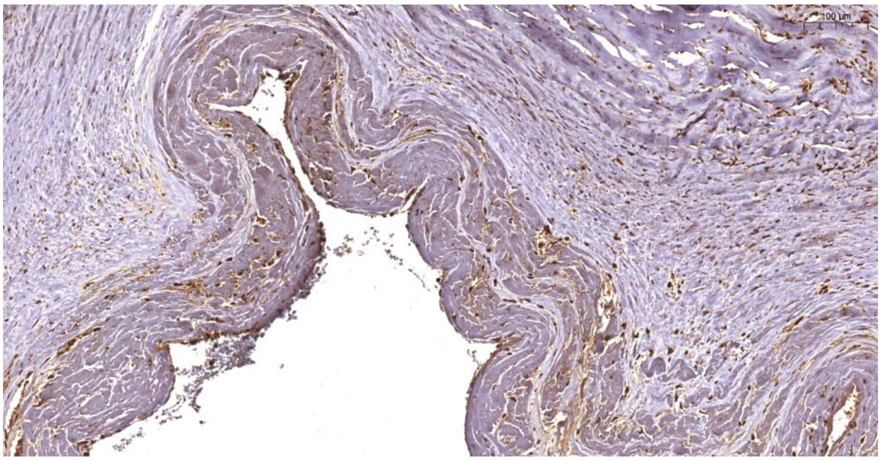

Supplementary material Figure 1. Immunostaining of CARD8 in non-atherosclerotic arteries (original picture)

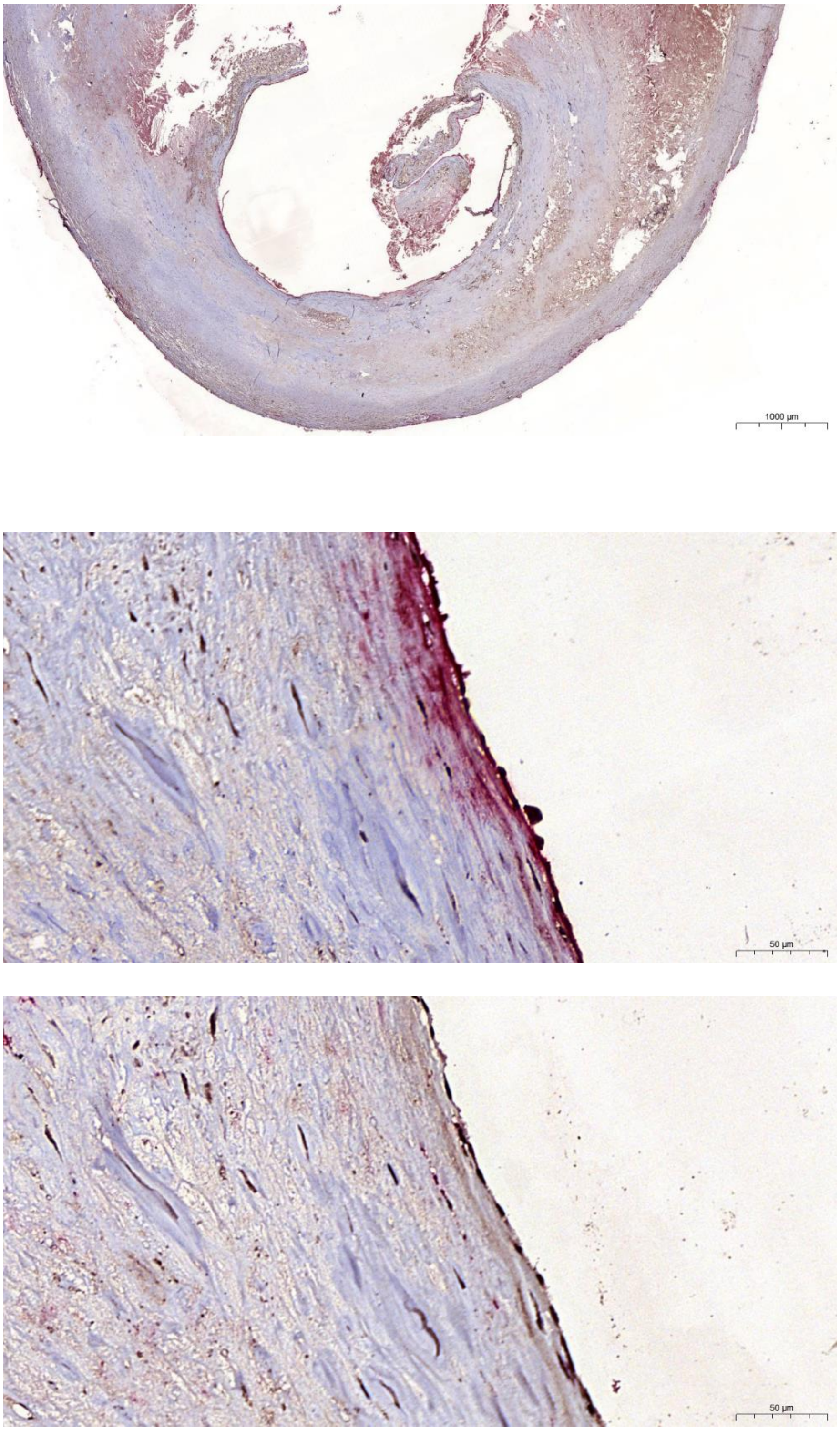

Supplementary material Figure 2 A. Representative image of immunostaining of human carotid atherosclerotic plaque (original picture)

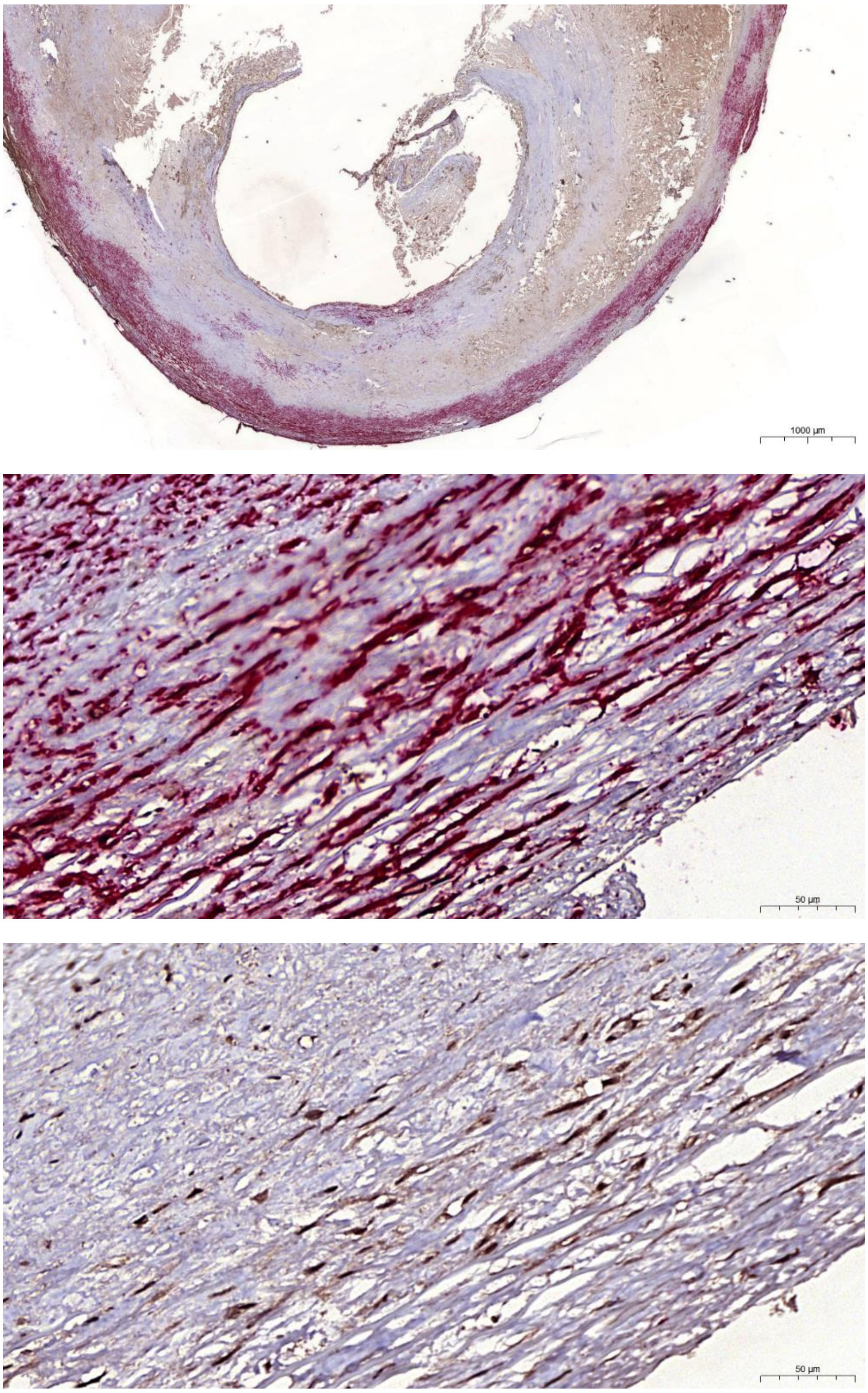

Supplementary material Figure 2 B. Representative image of immunostaining of human carotid atherosclerotic plaque (original picture)

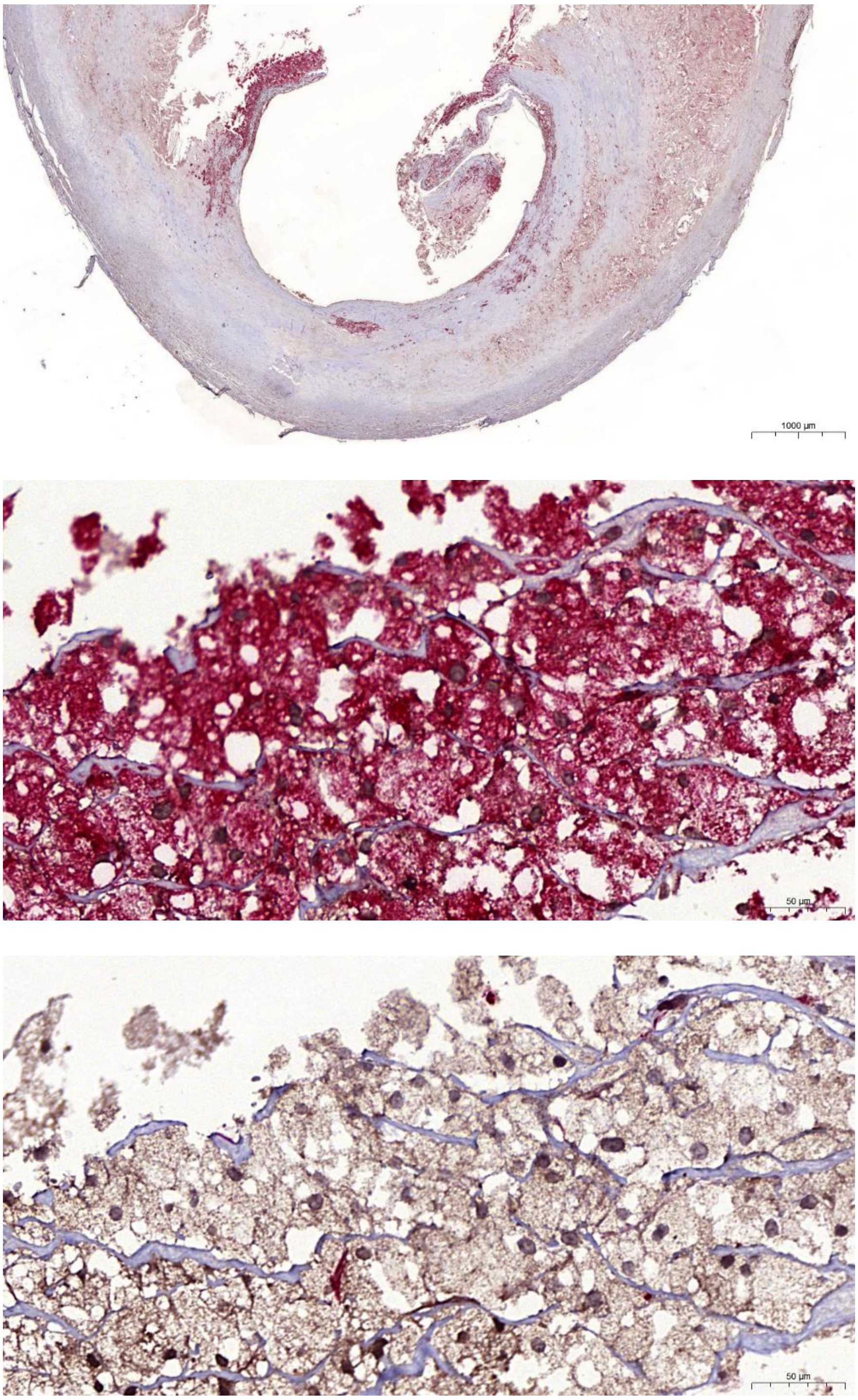

Supplementary material Figure 2 C. Representative image of immunostaining of human carotid atherosclerotic plaque (original picture)

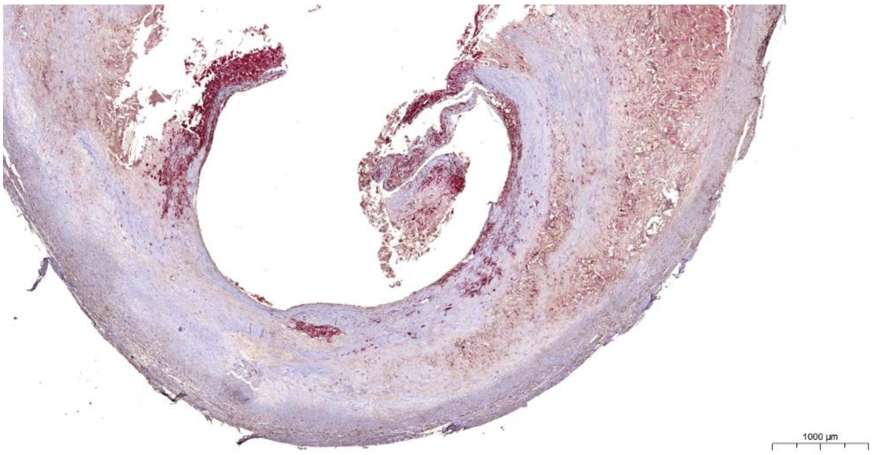

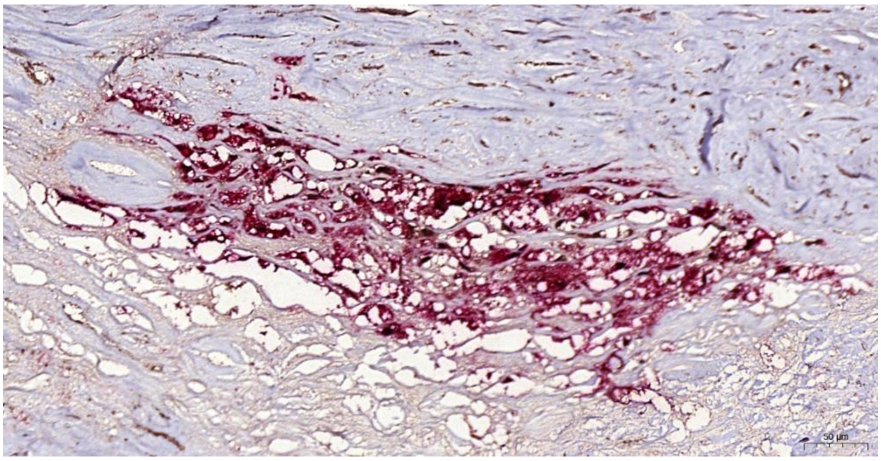

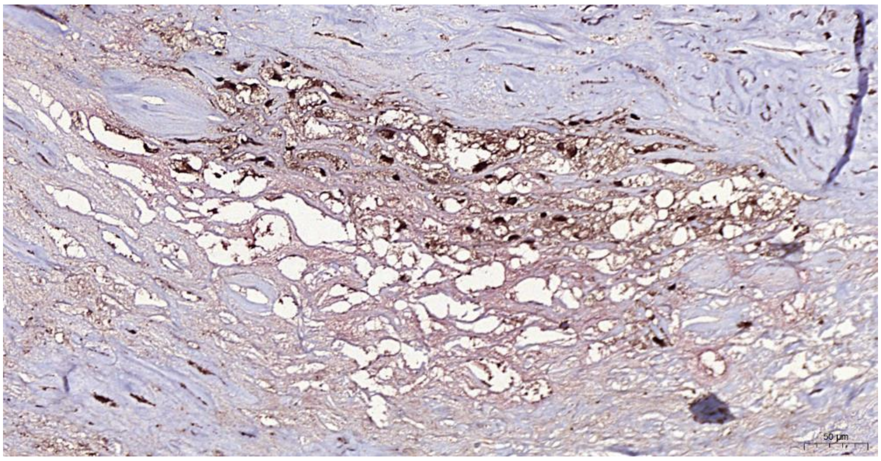

Supplementary material Figure 2 D. Representative image of immunostaining of human carotid atherosclerotic plaque (original picture)

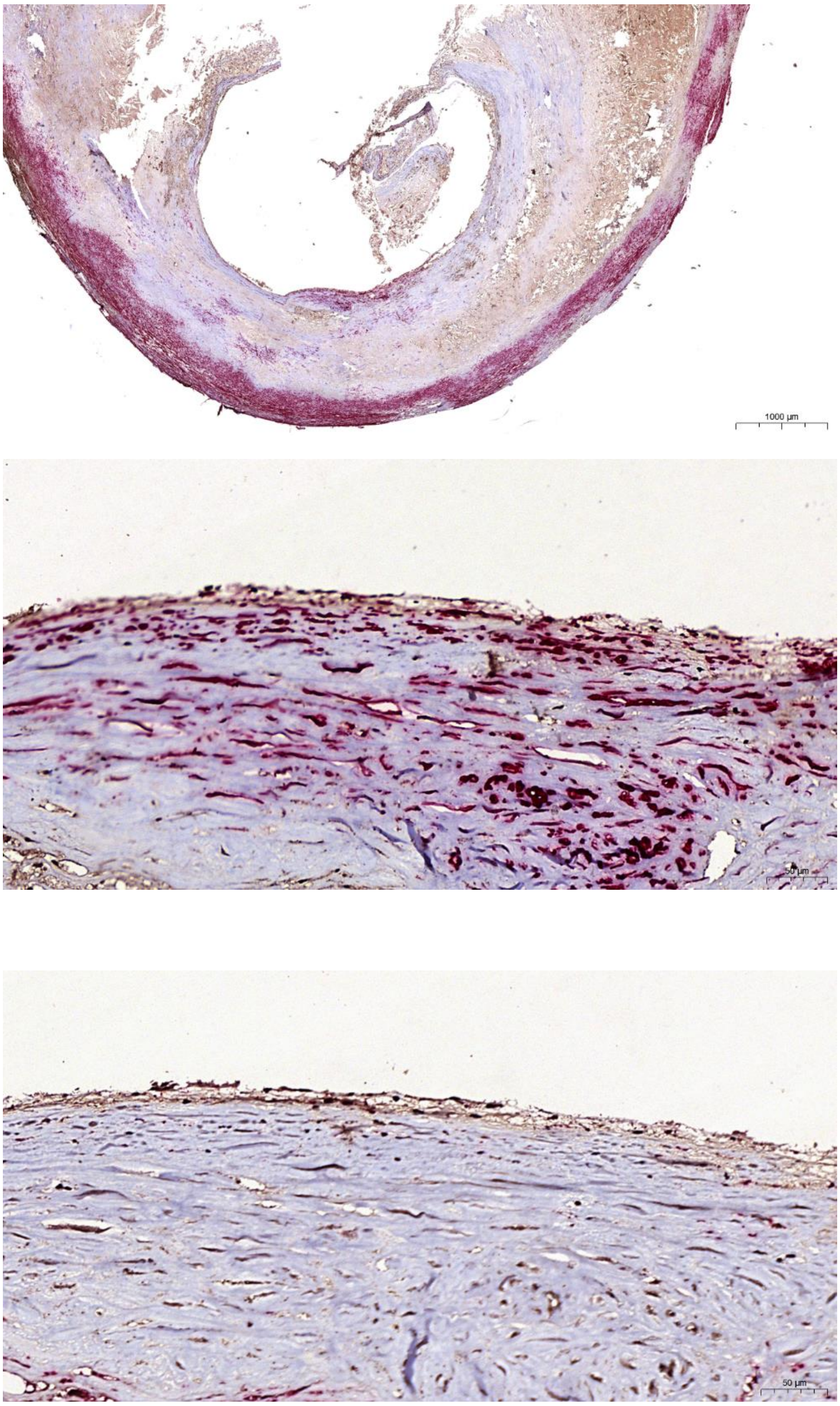

Supplementary material Figure 2 E. Representative image of immunostaining of human carotid atherosclerotic plaque (original picture)

Supplementary Figure 3 C: Uncropped Western blots for CARD8 and β-actin expression from repeated experiments. The membrane was developed for both the proteins simultaneously using species specific IRDye® Secondary Antibodies (Licor).

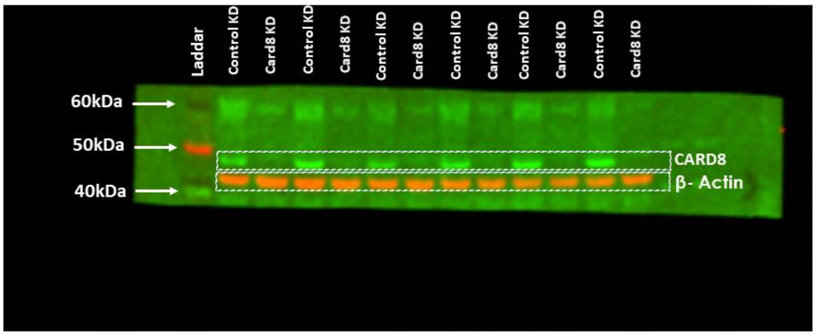

Supplementary material Figure 4: Expression of CARD8 mRNA and protein in control and *CARD8* knock down HUVECs. (full blot)

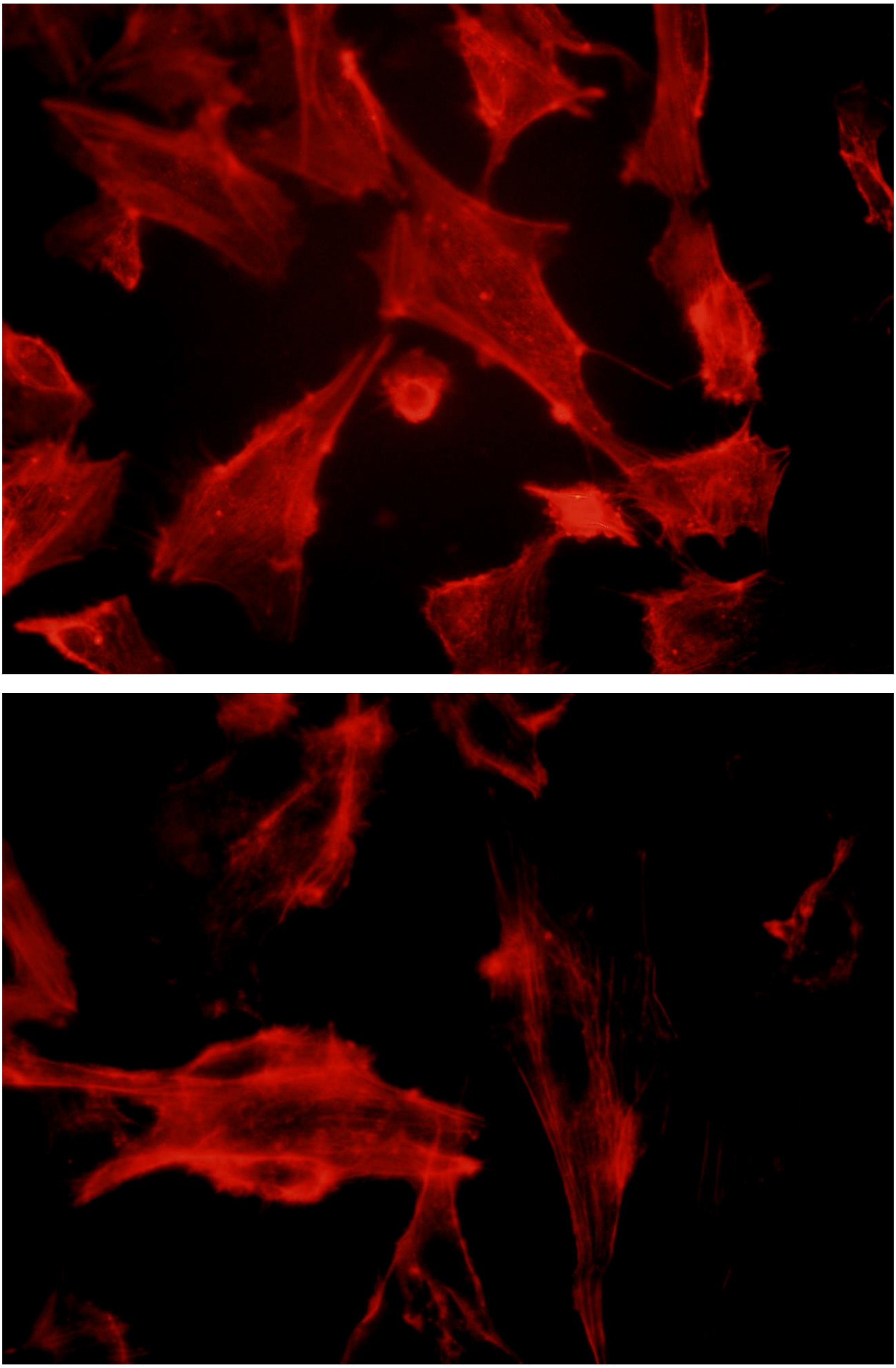

Supplementary material Figure 5. Subcellular localization of CARD8 in HUVEC. (Actin)

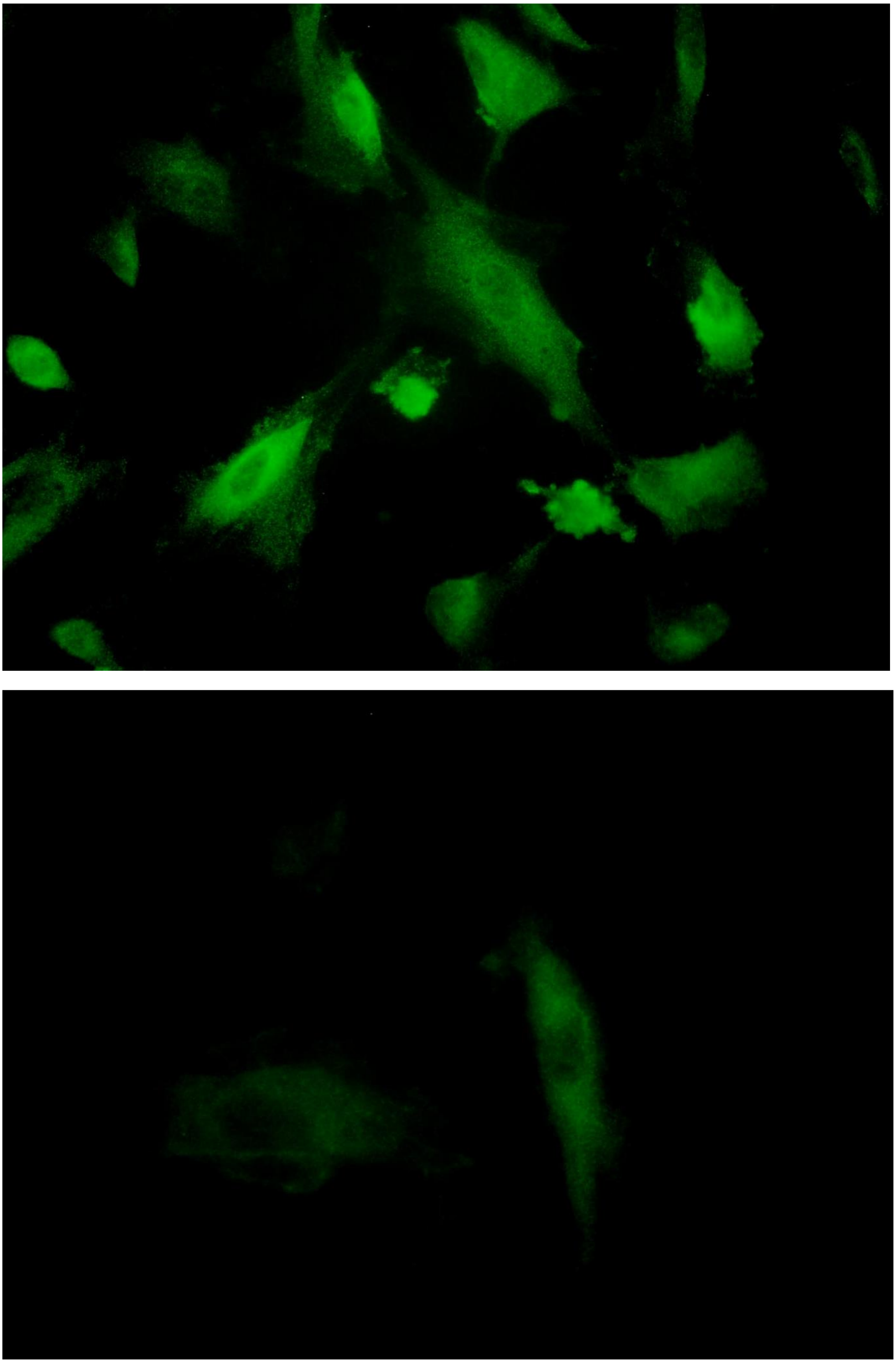

Supplementary material Figure 5. Subcellular localization of CARD8 in HUVEC. (CARD8)

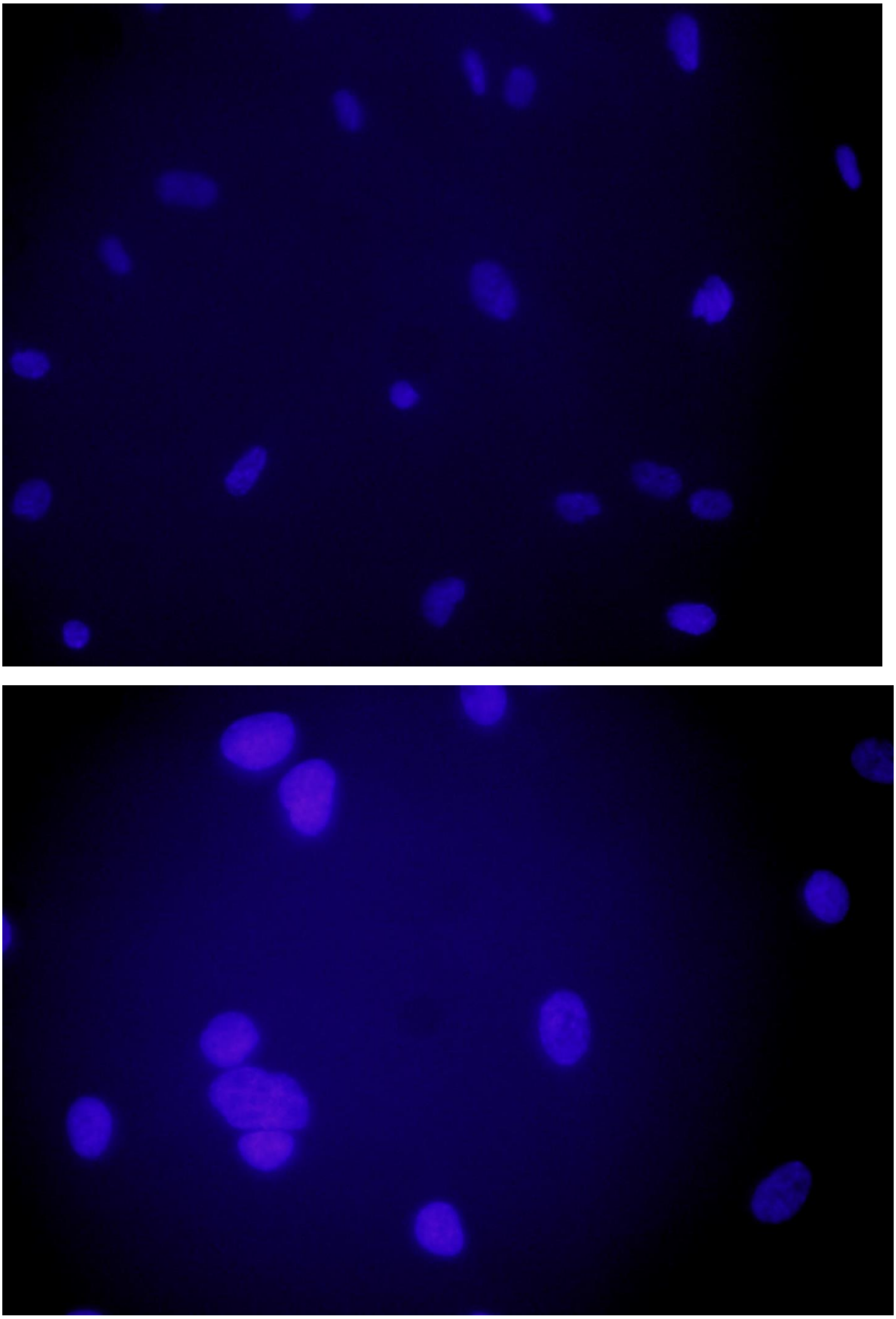

Supplementary material Figure 5. Subcellular localization of CARD8 in HUVEC. (DAPI)

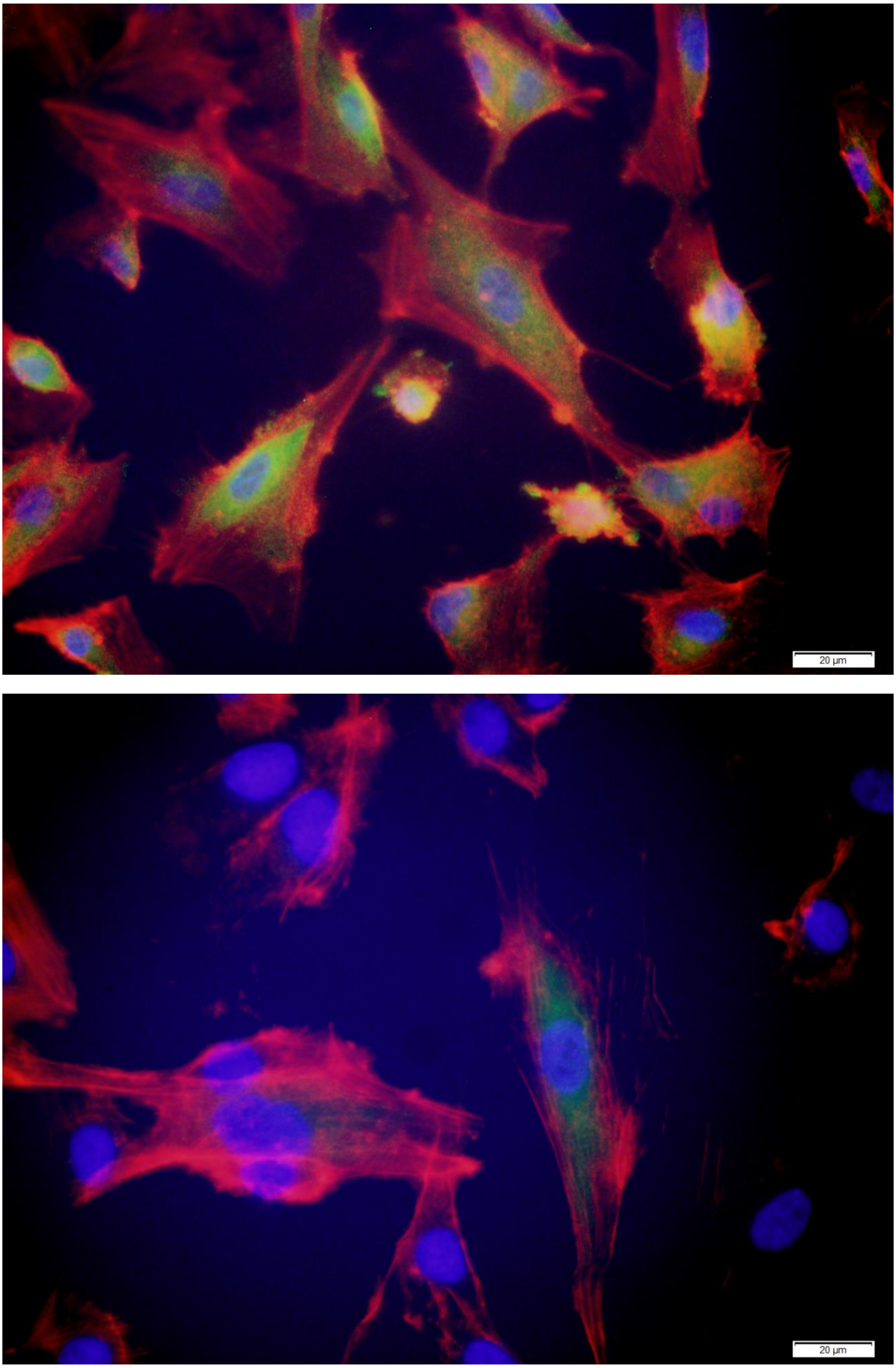

Supplementary material Figure 5. Subcellular localization of CARD8 in HUVEC. (Merge)
